# 3’UTR RNA editing driven by ADAR1 modulates MDM2 expression in breast cancer cells

**DOI:** 10.1007/s10142-025-01611-3

**Published:** 2025-05-17

**Authors:** Elanur Almeric, Deniz Karagozoglu, Mustafa Cicek, Didem Naz Dioken, Huseyin Avni Tac, Esra Cicek, Busra Aytul Kirim, Irmak Gurcuoglu, Osman Ugur Sezerman, Nurhan Ozlu, Ayse Elif Erson-Bensan

**Affiliations:** 1 Department of Biological Sciences, Middle East Technical University (METU), Dumlupinar Blvd. No.1 Universiteler Mah, Cankaya, Ankara, 06800 Türkiye; 2 Department of Biology, Kamil Ozdag Faculty of Science, Karamanoglu Mehmetbey University, Karaman, Türkiye; 3 School of Medicine, Department of Basic Sciences, Biostatistics and Medical Informatics, Acibadem Mehmet Ali Aydinlar University, Istanbul, Türkiye; 4 Department of Molecular Biology and Genetics, Koc University, Rumelifeneri Yolu, Sariyer, Istanbul, 34450 Türkiye; 5 Cancer Systems Biology Laboratory, CanSyL, METU, Ankara, Türkiye

**Keywords:** RNA editing, 3’UTR, MDM2, GINS1, F11R, ADAR1, CSTF2, Proximity biotinylation

## Abstract

Epitranscriptomic changes in the transcripts of cancer related genes could modulate protein levels. RNA editing, particularly A-to-I(G) editing catalyzed by ADAR1, has been implicated in cancer progression. RNA editing events in the 3’ untranslated region (3’UTR) can regulate mRNA stability, localization, and translation, underscoring the importance of exploring their impact in cancer. Here, we performed an in silico analysis to detect breast cancer enriched RNA editing sites using the TCGA breast cancer RNA-seq dataset. Notably, the majority of differential editing events mapped to 3’ untranslated regions (3’UTRs). We confirmed A-to-I(G) editing in the 3’UTRs of *MDM2* (Mouse Double Minute 2 homolog), *GINS1* (GINS Complex Subunit 1), and *F11R* (Junctional Adhesion Molecule A) in breast cancer cells. RNA immunoprecipitation with ADAR1 antibody confirmed the interaction between ADAR1 and *MDM2*, *GINS1*, and *F11R* 3’UTRs. ADAR1 knockdown revealed decreased editing levels, establishing ADAR1 as the editing enzyme. A reporter assay for *MDM2*, an oncogene overexpressed mostly in luminal breast cancers, demonstrated that RNA editing enhances protein expression, in agreement with reduced MDM2 protein levels in ADAR1 knockdown cells. Further exploration into the mechanisms of 3’UTR editing events revealed an interaction between ADAR1 and CSTF2, a core component of the polyadenylation machinery, as identified through biotin-based proximity labeling mass spectroscopy, and co-immunoprecipitation experiments. Furthermore, CSTF2 knockdown reduced both ADAR1 and MDM2 protein levels. Our findings highlight implications for *MDM2* regulation by ADAR1-dependent 3’UTR RNA editing and present an interplay between RNA editing on 3’UTRs and the mRNA polyadenylation machinery. These results improve our understanding of ADAR1’s role in cancer-associated 3’ UTR RNA editing and its potential as a therapeutic target.

**Supplementary Information:**

The online version contains supplementary material available at 10.1007/s10142-025-01611-3.

## Introduction

The mammalian transcriptome is highly diversified through various mechanisms, including RNA modifications. RNA editing by ADAR enzymes is one of the most prominent examples of widespread RNA modifications. RNA editing involves the deamination of adenosine to inosine or cytosine to uracil. Adenosine-to-inosine (A-to-I) RNA editing is catalyzed by adenosine deaminases (i.e., ADARs), subsequently leading inosines to be recognized as guanosines due to their similar electrostatic potential (Bass 2002). As a result, A-to-I editing within coding regions can modify codons, resulting in the production of altered protein isoforms (Maas et al., 2006). In untranslated regions (UTRs), RNA editing can influence RNA stability, localization, translation and modulate interactions with trans-factors (Mendoza et al., 2024).

ADAR1 (ADAR) is widely expressed across the different organs of the body as the principal A-to-I RNA editor and exists in two isoforms: the interferon-inducible p150 and the constitutively expressed p110. ADAR1 mediates editing by binding to double-stranded RNA (dsRNA) regions, which are commonly formed by inverted repeats, such as *Alu* elements. However, secondary structures within non-repetitive sequences can also serve as editing substrates (Liu et al. 2024). ADAR2 (ADARB1) is predominantly expressed in the brain, editing transcripts expressed in the central nervous system. ADAR3 (ADARB2) is a brain-specific but catalytically inactive enzyme (Rehwinkel and Mehdipour 2025).

Deregulated RNA editing has been implicated in various diseases, including neurodevelopmental disorders, autoimmune conditions, and cancer (Bass 2002). Notably, advancements in RNA sequencing technologies have brought increasing attention to the widespread nature and role of A-to-I(G) modifications in cancer. Numerous studies have shown that both ADAR1 levels and editing levels are markedly altered in many malignant tumors, with strong correlations to tumor progression and proteomic diversity in cancer (Zhang et al. 2024; Peng et al. 2018). However, the role of RNA editing in the non-coding regions of cancer-related genes remains to be further investigated.

In this study, we investigated ADAR1-mediated A-to-I(G) RNA editing events in breast cancer, with a focus on 3’UTRs. Using TCGA RNA-seq data, we identified differentially edited 3’UTRs in breast cancers and experimentally validated editing sites in the 3’UTRs of *MDM2*, *GINS1*, and *F11R* regulated by ADAR1 with protein level implications. We also provide insight into ADAR1 interaction with the polyadenylation machinery, supporting the possibility that ADAR1 may associate with transcripts at or near their 3’UTRs. These findings underscore ADAR1’s regulatory role in cancer and its potential as a therapeutic target.

## Methods

### In silico analysis

RNA editing regions in tumor (*n* = 837) and normal samples (*n* = 105) of the TCGA Breast Cancer RNA-seq were taken from the Synapse database (SynID: syn2374375) (https://www.synapse.org/) (Han et al. 2015). RNA editing sites which were present in at least 5 pairs of tumor and non-tumor samples were selected as informative RNA editing sites. Wilcoxon test was used to detect differential editing between tumor and non-tumor samples. Significantly different editing sites were defined with FDR < 0.05 and a mean editing level difference ≥ 5% between tumor and non-tumor samples. RNA editing sites were annotated using R package “Annotatr” with human genome assembly GRCh37 (hg19) and were matched to corresponding gene IDs. An UpSet plot was generated using the Python upsetplot package to visualize the intersection of editing regions on single mRNAs to identify patterns of possible co-occurrence.

### Cell lines and growth conditions

MCF7, T47D and MDA-MB-231 breast cancer cells and cells were cultured in Dulbecco’s Modified Eagle Medium (DMEM) supplemented with 10% Fetal Bovine Serum (FBS) (Biowest, S1810-500), 2% L-Glutamine, 1% Sodium Pyruvate Solution and 1% Penicillin/Streptomycin (BI, 03-031-1B). Cells were incubated at 37 °C with 95% humidified air and 5% CO_2_ and were grown as monolayers. Normal breast tissue cDNA was used from Breast Cancer cDNA Array IV (Origene).

### DNA, RNA isolation and RT-PCR

Genomic DNA (gDNA) was isolated from cell pellets using the lysis buffer (Tris-HCl (pH 8.5), EDTA, NaCl, and SDS). Following proteinase K and RNase A treatments, DNA was precipitated with ethanol and sodium acetate. Total RNA was extracted using the High Pure RNA Isolation Kit (Roche, 11828665001). RNA samples were DNase-treated (Thermo Fisher Scientific, EN0521) to eliminate DNA contamination. DNA and RNA quantity and purity were assessed using a MaestroNano spectrophotometer. cDNA was synthesized using the RevertAid First Strand cDNA Synthesis Kit (Thermo Fisher Scientific, K1622) using oligo(dT) primers. RT-PCR primers were designed from regions flanking the editing sites. PCR amplification was performed using Phusion™ High-Fidelity DNA Polymerase (Thermo Fisher Scientific, F530S). Following gel electrophoresis, PCR amplicons were extracted using Monarch^®^ DNA Gel Extraction Kit (NEB, T1020) according to the manufacturer’s protocol and sequenced using MiSeq NGS System (Illumina).

### Reporter assay

Edited and non-edited regions were designed as double-stranded oligos. Annealed oligos were cloned into pMIR-Report (pMIR). Cells were co-transfected with pMIR (*Firefly* luciferase) and phRL-TK (*Renilla* luciferase) using TurboFect (Thermo Fisher). Twenty-four hours after transfection, dual luciferase activities were measured using the Dual-Luciferase^®^ Reporter Assay (Promega). Firefly/Renilla ratios were normalized to that of the empty pMIR transfected cells.

### ADAR1 and CSTF2 silencing

FlexiTube GeneSolution for ADAR1 (QIAGEN, GeneGlobe Id: GS103, 1027416), CSTF2 FlexiTube GeneSolution for CSTF2 (QIAGEN, Gene Globe Id: GS1478, 1027416), and negative control siRNA (5 nmol) (QIAGEN, 1022076) were used. A 50 nM siRNA cocktail was prepared with 200 µl of DMEM with 4500 mg/L high glucose and 4 µl TurboFect Transfection Reagent (Thermo-Scientific/R0531).

### RNA immunoprecipitation

RNA Immunoprecipitation (RIP) protocol was performed as described (Peritz et al. 2006). Approximately, 9 × 10⁶ MCF7 cells were lysed in polysome lysis buffer. For RIP, ADAR1 antibody (Abcam, ab168809), or normal rabbit IgG control (Cell Signaling, 2729) and Protein A/G magnetic beads were used. Following RNA extraction with phenol-chloroform-isoamyl alcohol, RNA concentrations were determined. cDNA was synthesized using the ProtoScript^®^ First Strand cDNA Synthesis Kit (NEB, E6300S) with d(T)23VN primers.

### CSTF2-TurboID plasmid

CSTF2 coding sequence (NM_001325.2) was retrieved from NCBI and cloned into TurboID_HA-pcDNA 3.1. TurboID vector was a kind gift from Prof. M. Muyan (METU). The cloned plasmid was confirmed by sequencing.

### Proximity-dependent biotinylation

CSTF2_TurboID_HA or just TurboID_HA (empty vector) constructs were transfected into MCF7 cells. After 48 h, cells were transfected with CSTF2_TurboID or TurboID control plasmid. 24 h later, CSTF2-TurboID transfected cells were either treated with 100 nM E2 (17β-Estradiol, Sigma 1250008) or ethanol. CSTF2_TurboID_HA-transfected cells were also treated with 50 µM Biotin (D-Biotin, Sigma-Aldrich, B-4639), and 1 mM ATP (Sigma-Aldrich, adenosine 5’-triphosphate disodium salt hydrate, A2383) for 3 h. Nuclear and cytoplasmic lysates were isolated and used for western blot analysis to determine fusion protein expression and biotinylation status using anti-HA (Abcam, ab9110), anti-ADAR1 (Abcam, ab168809), anti-CSTF2 (Abcam, ab72297), and anti-Biotin (Abcam, ab53494), anti-HDAC (Santa Cruz, sc-81598) and anti-TUBA1A (Cell Signaling, 2144) primary and appropriate secondary antibodies.

### Mass spectrometry analysis of TurboID samples

To identify prey proteins, (nuclear and cytoplasmic) biotinylated proteins bound to Neutravidin High-Capacity Agarose beads (Thermo Fisher Scientific, 29204) were sent to Koc University Proteomics Facility (KUPAM) for Liquid Chromatography–Mass Spectrometry (LC-MS/MS) analysis. Nuclear and cytoplasmic fractions were separately analyzed. Proteins bound to the beads were digested with trypsin (Thermo Pierce MS Grade Trypsin Protease), and peptide purification was performed using C18 StageTips (Thermo Fisher Scientific). Peptide analysis was conducted with the Q-Exactive Orbitrap LC-MS/MS mass spectrometer (Thermo Fisher Scientific), and protein identification was carried out using the Proteome Discoverer 1.4 software (Thermo Fisher Scientific). Any cytoplasmic protein was removed the nuclear lysate list and was not included in further analysis. SAINT (Significance Analysis of INTeractome) was used as a statistical tool to estimate the probability of protein-protein interactions using default parameters, comparing E2 treated and control cell nuclear samples (Choi et al. 2011). Proteins with a SAINT score greater than 0.5 and a BFDR (Bayesian False Discovery Rate) lower than 0.05 were considered significant.

### Co-immunoprecipitation

Nuclear lysates were prepared from MCF7 cells using NE-PER™ Nuclear and Cytoplasmic Extraction Kit (Thermo Fisher Scientific). Protein A/G magnetic beads (New England BioLabs) were blocked overnight with BSA, washed, and incubated with primary antibodies (CSTF2, Santa Cruz sc-398862 or mouse IgG control, Santa Cruz sc-202) and protein lysates. Beads were washed, and bound proteins were eluted with SDS loading buffer and analyzed by immunoblotting using CSTF2 (Abcam, ab72297) and ADAR1 (Cell Signaling, 14175) antibodies. Blots were visualized using WesternBright ECL Blotting Substrates (Advansta-K12045-D50) on a ChemiDoc™ MP Imaging System (Bio-Rad, 170–8280).

### *MDM2* mRNA and protein expression analysis

Log2-transformed RSEM TPM values (tumor, adjacent normal, and GTEx normal tissue) were downloaded from the TCGA Target GTEx study using the UCSC Xena platform. TCGA TARGET GTEx cohort was used. GTEx data was included to investigate whether histologically normal tissue adjacent to tumors had different expression levels, as previously indicated (Aran et al. 2017). Clinical data were obtained using the R package TCGAbiolinks and GDC Data Portal. For patients whose breast cancer subtypes were not reported in the GDC legacy clinical data, additional subtype information was obtained from cBioPortal using the other TCGA legacy datasets.

### Relapse free survival

Disease-free months and relapse events were downloaded from cBioPortal (TCGA BRCA GDC Legacy). Patients were stratified into high and low *MDM2* expression groups using the “auto select best cutoff” feature of the KMplotter (Győrffy 2021) where clinical and expression data were uploaded. Log2-transformed RSEM TPM values were downloaded from the TCGA Target GTEx study using the UCSC Xena platform. Patients with relapse-free survival times of 0 months or greater than 120 months (10 years) were excluded from the analysis.

## Results

### 3’UTR editing in breast cancers

**Fig. 1:**
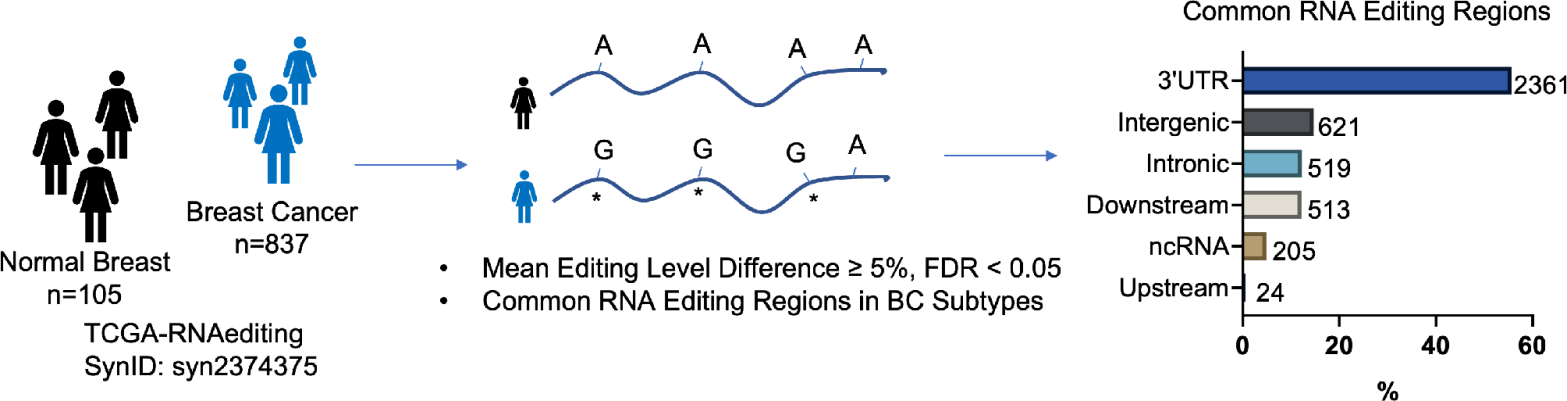
TCGA breast cancer (*n* = 837) and normal breast tissue (*n* = 105) RNA-seq data was screened for A-to-I(G) editing events. Informative RNA editing sites were identified in at least five tumor and non-tumor sample pairs. Differential editing was assessed using the Wilcoxon test, with significant sites defined by FDR < 0.05 and a mean editing level difference ≥ 5% between tumor and non-tumor samples. Majority of significant editing sites map to 3’UTRs. The upstream and downstream regions were defined as 1 kilobase (kb) sequences flanking the annotated transcription start and end sites of the gene, respectively. ncRNA: Noncoding RNA

Using the RNA editing sites defined in TCGA Breast Cancer RNA-seq dataset (TCGA-RNAediting, SynID: syn2374375) (Han et al. 2015), we detected differentially edited mRNAs in breast tumors (Fig. 1). Given that majority of differentially edited regions mapped to 3’UTRs, we focused on these editing events detected in all breast cancer subtypes (Table S1, Fig.S1a).

We started by experimentally confirming the A-to-I editing events in the 3’UTRs of selected transcripts based on their cancer relevance and high expression in breast tumors (Fig. S1b). Inosine (I) in the RNA is recognized as guanosine (G) by reverse transcriptase (RT), and therefore cytosine (C) is incorporated into the reverse-transcribed cDNA. Next, upon PCR amplification of the cDNA, DNA polymerase incorporates G into the new strand. Consequently, A-to-I editing is observed as a complete or partial replacement of edited A with G in the cDNA (Malik et al. 2021).

**Fig. 2:**
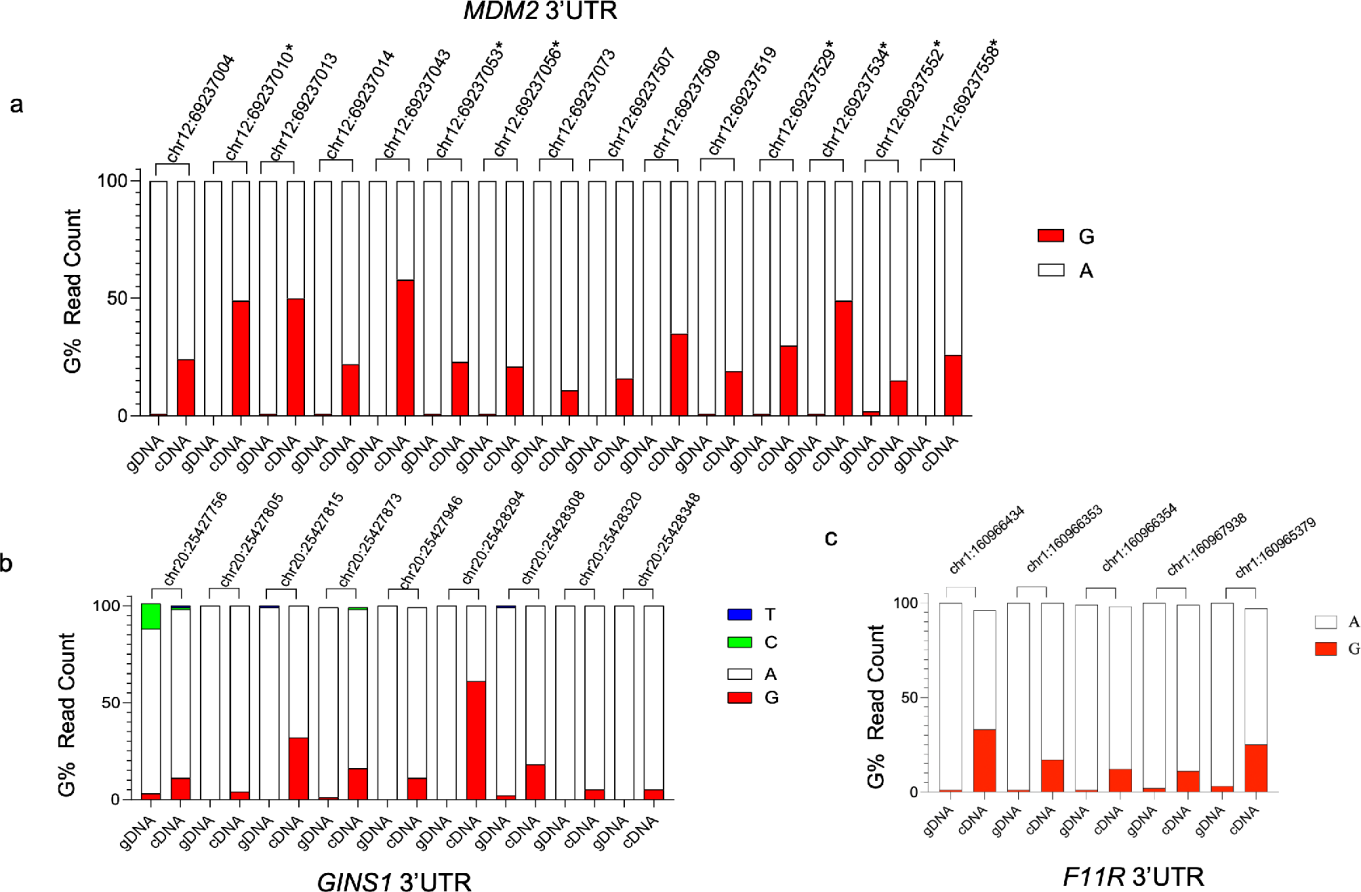
A-to-I(G) editing events in the 3’UTRs of *MDM2* (**a**), *GINS1* (**b**), and *F11R* (**c**) in MCF7 cells. gDNA (genomic DNA) or cDNA templated PCR amplicons were sequenced and G% values were calculated based on NGS read counts. The NGS confirmed A-to-I(G) editing events that were not initially predicted are indicated by *

To this end, we used primers (Table S2) flanking the editing sites and generated PCR amplicons by using genomic DNA or cDNA as templates. Following amplicon sequencing, we confirmed A-to-I(G) changes in the 3’UTRs of *MDM2* (mouse double minute 2 homolog), *GINS1* (GINS complex subunit 1), and *F11R* (junctional adhesion molecule A) (Fig. 2a, b, c, Table S3). A-to-I(G) editing sites were quantified by calculating G count percentage in RNA-seq reads at positions where an A was present in the gDNA templated amplicons (Malik et al. 2021). The initial editing predictions for *MDM2*, *GINS1*, and *F11R*, obtained from the TCGA breast cancer patient dataset was experimentally validated (Fig. 2). A few additional A-to-I(G) editing events were identified for *MDM2* that were not initially predicted (Fig. 2a, indicated by *). Importantly, none of these edit sites were reported as SNPs based on the DBSNP (The Single Nucleotide Polymorphism Database). We also checked the Database of RNA Editing in Humans (DARNED), further confirming that these sites were edited in diverse tissues (Table S4-6). Among the editing sites we confirmed, chr12:69237004, chr12:69237010, chr12:69237013, and chr12:69237053 have been previously shown to be edited in breast cancer, 12 of 15 sites for *MDM2*, 8 of 9 sites for *GINS1*, 4 of 5 sites for *F11R* of editing sites were also confirmed in lymphoblastoid cells according to DARNED (Bahn et al. 2012; Ramaswami et al. 2012). Moreover, independently amplified and sequenced PCR amplicons yielded comparable editing percentages in MCF7 cells, supporting the reproducibility of the results (Fig. S2a). Editing was further confirmed in MDA-MB-231 cells representing triple negative breast cancer cells (Fig. S2b-d).

### ADAR1 interaction with the 3’UTRs of *MDM2*,* GINS1* and *F11R*

**Fig. 3:**
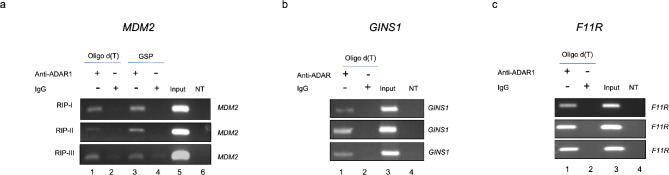
RIP-PCR for *MDM2* (**a**), *GINS1* (**b**) and *F11R* (**c**). For RNA-IP, ADAR1 (Abcam, ab168809), and rabbit IgG (Cell Signaling, 2729) antibodies were used. cDNAs were synthesized from RNA samples isolated after RIP with oligo d(T) primers (lanes 1, 2), or gene-specific primers (GSP) for *MDM2* (lanes 3,4). Three independent biological replicates are shown for each RIP experiment and PCR. Lane 5 for *MDM2*, and lanes 3 for *GINS1* and *F11R* RIP-PCRs had MCF7 cDNA (Input) as positive control for PCR. NT lane was no template/negative control

Next, to functionally test the relationship between ADAR1 and edited mRNAs, we performed ADAR1 immunoprecipitation of interacting RNAs and RT-PCR (RIP-PCR) to determine whether ADAR1 interacts with *MDM2*, *GINS1*, and *F11R* mRNAs. cDNAs were synthesized from ADAR1 immunoprecipitated RNAs using oligo (dT) or gene-specific primers. Editing events were examined in the MCF7 breast cancer model, representing the most common type of breast cancer (luminal A subtype), expressing estrogen receptor (ER) and/or progesterone receptor (PR) (Perou et al. 2000; Sorlie et al. 2003). Our results demonstrated that all three mRNAs, *MDM2*, *GINS1*, and *F11R* were specifically enriched in ADAR1 immunoprecipitation samples. In contrast, these mRNAs were not detected in the control samples immunoprecipitated with non-specific control IgG antibody (Fig. 3).

**Fig. 4:**
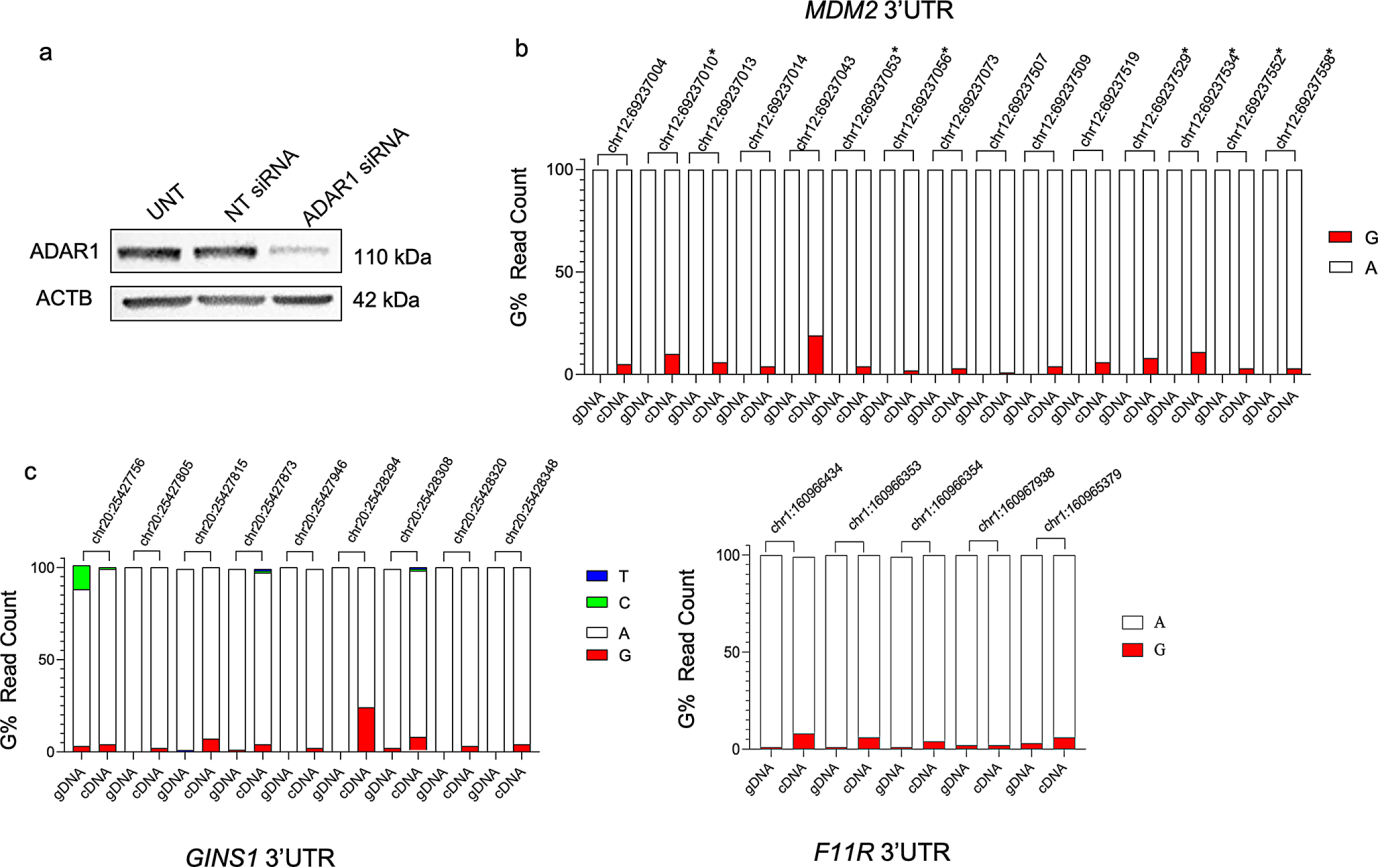
siRNA-mediated ADAR1 knockdown and RNA editing in MCF7 cells, **a**. Western blot shows ADAR1 protein levels in ADAR1 siRNA or NT (non-targeting) siRNA transfected cells. UNT: Untransfected cells. The same blots were hybridized with ACTB antibody to test sample loading, **b**. A-to-I(G) editing ratios of *MDM2*, **c**. A-to-I(G) editing ratios of *GINS1*, and A-to-I(G) editing ratios of *F11R* in ADAR1 knockdown MCF7 cells

We also sought to investigate whether these interactions were functional and knocked down ADAR1 using RNAi and sequenced RT-PCR amplicons covering the previously edited regions for *MDM2*, *GINS1*, and *F11R*. All three mRNAs, *MDM2*, *GINS1*, and *F11R* had markedly decreased editing levels compared to ADAR1-expressing cells. MDM2 editing levels were similar in NT siRNA transfected cells and untransfected MCF7 cells (Fig. 4, Fig. S2e).

To this end, based on in silico editing predictions from tumors, we were able to experimentally confirm editing events in the 3’UTRs of selected candidates, showing their physical and functional relationship with ADAR1. Next, given its established role in cancer, we focused on RNA editing events in the 3’UTR of *MDM2*.

### *MDM2* overexpression and editing in breast cancer

**Fig. 5:**
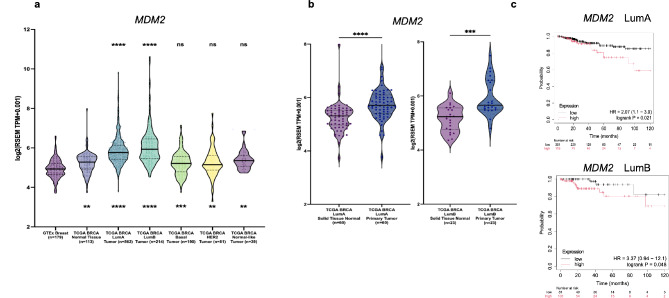
**a**. *MDM2* mRNA expression levels (log2-transformed RSEM TPM values (TPM + 0.001) in GTEx breast normal tissue (n = 179), TCGA breast cancer normal tissue (n = 113), and TCGA breast cancer tumor subtypes (luminal A (lumA,0 n = 562), luminal B (lumB, n = 214), basal (n = 190), HER2-enriched (n = 81), and normal-like (n = 39)). Asterisks above the violin plots indicate statistical significance compared to TCGA normal breast tissue data, while asterisks below the violin plots indicate statistical significance compared to GTEx normal breast tissue. One-way ANOVA with Tukey correction for multiple comparisons was performed (**** p < 0.0001, *** p < 0.001, ** p < 0.01, and “ns” for non-significant), **b**. *MDM2* expression in lumA and lumB breast tumors compared with matched normal tissue (paired t-test, **** p < 0.0001), **c**. Kaplan-Meier analysis of relapse-free survival (RFS) in TCGA BRCA lumA and lumB patients based on *MDM2* transcript levels. Patients were grouped into ‘high’ (red) and ‘low’ (black) expression categories based on “Auto select best cutoff” option using data upload. For lumA, Hazard ratio (HR) is 1.98 (95% CI: 1.06–3.71) with a log-rank p-value of 0.021. For lumB, log-rank p-value is 0.048 (HR:3.37). The number of patients at low vs high risk at different time points is indicated below the graphs

First, we determined *MDM2* levels in breast cancers using GTEx normal breast tissue (*n* = 179), TCGA normal breast tissue (*n* = 113), and TCGA breast cancer tumor subtypes (luminal A (*n* = 562), luminal B (*n* = 214), basal (*n* = 190), HER2-enriched (*n* = 81), and normal-like (*n* = 39). Curiously, *MDM2* is most significantly overexpressed in luminal A (lumA) breast cancers in the TCGA dataset compared with normal breast tissue or adjacent normal tissue (Fig. 5a, b). For lumA tumors, high *MDM2* transcript levels correlate with poor relapse-free survival times (*p* < 0.05) (Fig. 5c). In line with these findings, an earlier study suggested MDM2 as an independent negative prognostic marker for breast cancer based on IHC performed in more than 2000 breast carcinomas (Turbin et al. 2006).

**Fig. 6:**
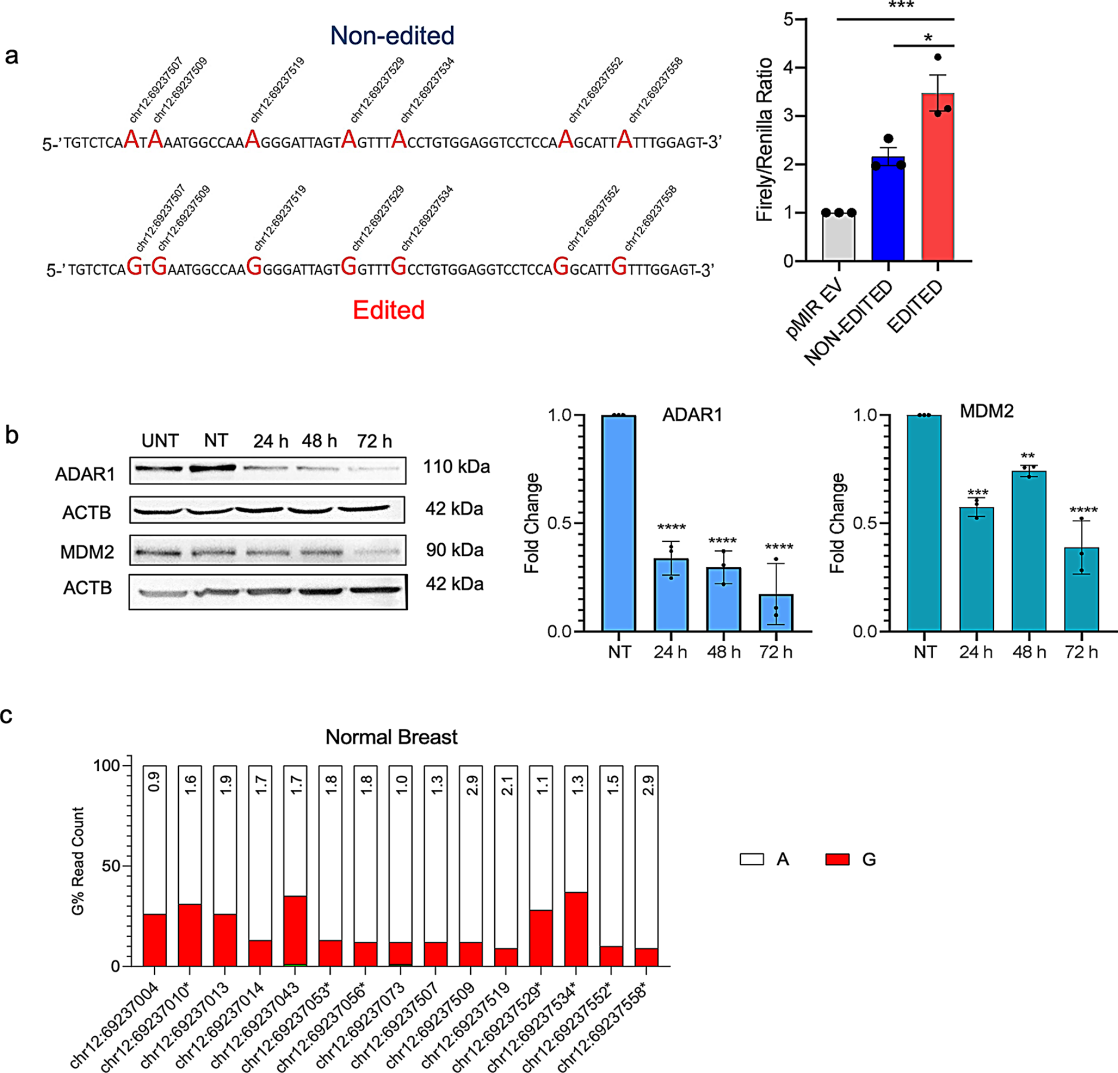
Effect of RNA editing on MDM2 protein levels, **(a)** Dual luciferase reporter assay with *MDM2* 3’UTR edit rich region. Non-edited (A) and edited (G) oligos were cloned downstream of the luciferase gene in pMIR. MCF7 cells were transiently transfected, and *Firefly/Renilla* luciferase read-outs from the constructs were normalized to that of empty pMIR (EV) (*p < 0.05, ***p < 0.0005, *n* = 3 independent transfections, one-way ANOVA, Tukey’s HSD), **(b)** ADAR1 and MDM2 protein levels in ADAR1 siRNA transfected (24 h, 48 h, 72 h) MCF7 cells. The same blots were hybridized with ACTB antibody to test sample loading. The image is representative of 3 independent experiments. Graphs show densitometric quantification of MDM2 and ADAR1 bands normalized to NT siRNA transfected cells (**p < 0.005, ***p < 0.0005, ****p < 0.0001, one-way ANOVA, Tukey’s HSD). NT: non-targeting siRNA transfected cells, UNT: Untransfected cells, **(c)** NGS and G% reads for the fifteen RNA editing positions for the 3’UTR of *MDM2* in normal breast tissue. Fold changes in RNA editing percentage values between MCF7 cells and normal breast cDNA are shown on the bars

*MDM2* has an unusually long 3’UTR, harboring previously described cis-elements recognized by trans-factors, including microRNAs (Zhang et al. 2016). To investigate the functional impact of RNA editing, we focused on the highly edited region within the *MDM2* 3’UTR. Double-stranded oligonucleotides were synthesized to mimic either the non-edited or edited state, where the edited oligo featured guanine (G) substitutions at the adenosine (A) positions for seven nucleotides within the highly edit-rich region of the *MDM2* 3’UTR. The edited and non-edited sequences were cloned downstream of the firefly luciferase coding sequence of the pMIR vector (Fig. 6a). Cells were co-transfected with pMIR and phRL-TK (*Renilla* luciferase) for normalization, and dual luciferase activity was measured (Fig. 6a). The edited region exhibited a 60% increase in luciferase activity compared to the non-edited 3’UTR fragment (*p* < 0.05), indicating that RNA editing positively influences MDM2 protein levels. Notably, this reporter assay provided insight into a short segment of the *MDM2* 3’UTR undergoing editing. Additional evidence supporting the role of RNA editing in modulating MDM2 protein levels was obtained from ADAR1 knockdown experiments. Reduced ADAR1 expression led to a significant decrease in A-to-I(G) editing ratio, and decreased MDM2 protein levels (Figs. 4b and 6b). These findings collectively suggest that 3’UTR RNA editing modulates MDM2 protein levels in cancer cells. Furthermore, supporting the cancer-specific nature of this editing, *MDM2* RNA editing was less prevalent in normal breast tissue (Fig. 6c, Table S7).

While these experiments offered preliminary insights into the functional relevance of several RNA editing sites within the *MDM2* 3′UTR, a more comprehensive analysis using full-length 3’UTR constructs, coupled with functional assays, would be necessary to determine whether RNA editing alters the binding affinity of microRNAs or other trans-acting regulatory factors. Supporting this possibility, in silico target prediction identified putative microRNA binding sites in close proximity to the validated editing sites within the *MDM2* 3′UTR (Fig. S3a).

### ADAR1 in close proximity to the polyadenylation machinery

**Fig. 7:**
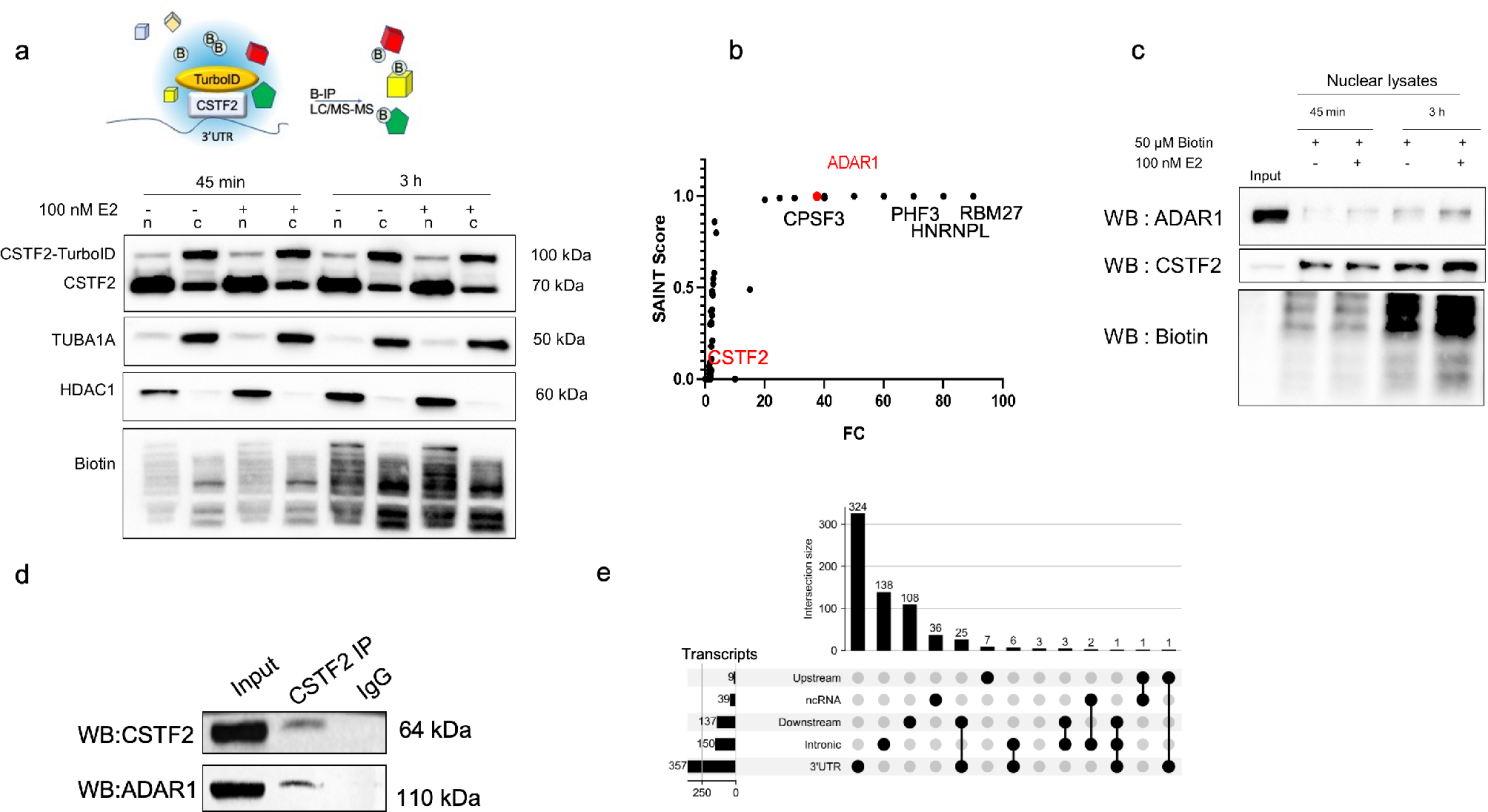
ADAR1 interacts with CSTF2, **a.** Schematic shows the proximity biotinylation approach to detect protein interactions at 3’UTRs using CSTF2_TurboID_HA fusion protein expression and biotinylation in MCF7 cells. Biotinylated target proteins were immunoprecipitated (B-IP) and analyzed by LC-MS/MS. Western blot analysis of CSTF2-TurboID transfected MCF7 cells treated with 100 nM E2 or ethanol for 45 min–3 h, and 50 µM biotin. Nuclear and cytoplasmic lysates were subjected to western blot analysis using anti-CSTF2 antibody to detect CSTF2_TurboID_HA fusion and endogenous CSTF2. HDAC1 and TUBA1A antibodies were used to validate the nuclear and cytoplasmic fractions. Anti-Biotin antibody was used for biotinylation assessment, **b.** Biotinylated nuclear proteins were identified through LC-MS/MS analysis. ADAR1 was found to be significantly enriched among the biotinylated nuclear proteins (SAINT score 1), **c.** ADAR1 was confirmed to be biotinylated by the CSTF2-TurboID fusion. MCF7 cells were transfected with CSTF2-TurboID. After 24 h, cells were incubated with 50 µM Biotin in the presence or absence of E2 for 45 min and 3 h. Isolated nuclear and cytoplasmic lysates were used for affinity capture with streptavidin beads. Eluted proteins were used for western blot analysis with anti-ADAR1, anti-CSTF2 and anti-Biotin antibodies, **d**. Nuclear extracts (500 µg) of MCF7 cells were subjected to Co-IP with CSTF2 or isotype-matched IgG. Input was 25 µg nuclear lysate. Immunoprecipitated proteins were then subjected to immunoblotting using CSTF2 or ADAR1 antibodies. WB: western blotting, **e.** Upset plot showing intersection of RNA editing events on mRNAs. Majority of RNA editing events were only detected on 3’UTRs. Each row represents a genomic feature, and the filled dots indicate the intersection of editing regions in individual mRNAs. The vertical bars indicate the number of genes in each category

Considering the predominance of RNA editing in the *Alu* regions (Fumagalli et al. 2015), we used RepeatMasker to investigate the presence of repeat elements around the A-I(G) sites. There were no *Alu* elements around MDM2 editing (Fig. S3b). Nevertheless, we sought to confirm the presence of ADAR1 in the 3’UTR region despite the lack of *Alu* elements. We hypothesized that RNA-binding proteins associated with 3’UTRs might interact with ADAR1 and play a role in region-selective editing. To investigate this, we selected CSTF2—a well-known RNA-binding protein associated with 3’ UTRs and a core component of the polyadenylation machinery—as a candidate to identify potential protein partners that might explain ADAR1’s presence on 3’ UTRs. We employed a proximity labeling approach for this analysis. We considered the dynamic nature of transcription and post-transcriptional mechanisms and used the TurboID-based proximity-labeling because of its faster kinetics compared to the original BirA biotin ligase (Roux et al. 2012). We transfected estrogen receptor-positive MCF7 cells with the CSTF2-TurboID construct and treated them with E2 (Estradiol) to stimulate transcription and proliferation (Ayaz et al. 2019). We confirmed the expression and biotinylation potential of CTSF2-TurboID by incubating the cells with 50 µM biotin for 3 h (Fig. 7a). Endogenous CSTF2 was mostly present in the nuclear fraction (n), whereas CSTF2-TurboID fusion protein was detected in both the nucleus and cytosol (c) (Fig. 7a). Of note, E2 treatment to enhance transcription and proliferation did not alter the subcellular localization of the fusion protein. Following this characterization, target proteins were labeled using the described conditions.

To capture nuclear targets of CSTF2-TurboID, biotinylated proteins in the nuclear and cytoplasmic lysates were separately captured using streptavidin-conjugated magnetic beads and subjected to LC-MS/MS analysis. The experiment was repeated twice, with two technical replicates for each sample (E2 or ethanol-treated). All biotinylated proteins in the cytoplasmic fraction were subtracted from the nuclear fraction and the SAINT algorithm was used to probabilistically score protein-protein interactions in E2 treated cells (Choi et al. 2011) (Fig. 7b). Of note, we did not capture any E2-spesific biotinylation of proteins by the CSTF2-TurboID construct. In addition to the expected interactors of CSTF2 that appeared as biotinylated proteins (e.g., CSTF2 itself, CPSF3, and CPSF2), 13 proteins had a SAINT score of 1. These candidates were considered high-confidence CSTF2 interactors (Table S8). Interestingly, ADAR1 emerged as one of the most significant nuclear interactors of CSTF2 (Saint Score 1, FC:40, BFDR:0) (Fig. 7b, Table S8). Consistent with this interaction, ADAR1 is predominantly localized in the nucleus (Fig. S4).

To confirm the LC-MS/MS results and explore a potential interaction between ADAR1 and CSTF2, we first performed an independent proximity biotinylation experiment. CSTF2-TurboID biotinylated proteins were pulled down and subjected to western blotting. CSTF2 and ADAR1 were both found in the biotinylated fraction (Fig. 7c). Using an independent approach, we performed Co-IP in untransfected MCF7 cells. Immunoprecipitation of nuclear extracts using the CSTF2 antibody followed by immunoblotting with the ADAR1 antibody confirmed the presence of ADAR1 in the immunoprecipitated lysates (Fig. 7d, Fig. S5 for biological replicates for the Co-IP experiment). These results suggest that ADAR1 interacts with CSTF2, a critical component of the polyadenylation machinery. In addition, we found that the majority of differentially edited mRNAs exhibited editing exclusively within their 3′UTRs, with no detectable editing in other transcript regions (Fig. 7e). This pattern may indicate a 3′UTR-specific accessibility or the selective recruitment of ADAR1 to these regions.

**Fig. 8:**
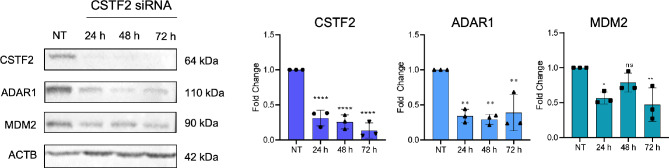
Effect of CSTF2 knockdown on ADAR1 and MDM2 protein levels. Western blot analysis of CSTF2 siRNA transfected MCF7 cells (24, 48, and 72 h) showed decreased ADAR1 and MDM2 protein levels. The same blots were hybridized with ACTB antibody to test sample loading. The image is representative of 3 independent experiments. Graphs show densitometric quantification of bands normalized to NT bands (ns: not significant, *p < 0.05, **p < 0.01, ****p < 0.0001; *n* = 3 biological replicates, one-way ANOVA, Tukey’s HSD). NT: non-targeting siRNA transfected cells

It remains unclear whether the interaction between ADAR1 and CSTF2 and/or other polyadenylation machinery components is essential for RNA editing at specific 3’UTRs, including *MDM2*. It is also not clear whether this interaction is direct and/or is RNA dependent. Possibly due to the essential role of CSTF2 in mRNA maturation, CSTF2 knockdown resulted in a 60% reduction in ADAR1, and 40–50% reduction in MDM2 protein levels, which precluded the ability to assess the role of the CSTF2-ADAR1 interaction in RNA editing under these conditions for *MDM2* (Fig. 8). Of note, siRNA-mediated knockdown of CSTF2 or ADAR1 in MCF7 cells did not result in a noticeable reduction of *MDM2* transcript levels and there was no correlation between *ADAR1 *and *MDM2 *mRNAs levels in the TCGA breast cancer RNA-seq data for LumA patients (Fig. S7). There is also no correlation between MDM2 and ADAR1 mRNA expression in Luminal A breast cancer samples (TCGA) (Fig. S7).

Overall, these findings highlight the impact of 3’UTRs and RNA editing on protein levels of MDM2 and potentially other cancer-related genes. Elucidating the mechanism of RNA editing for activation or inactivation of cancer-related genes may open new avenues for epitranscriptome targeted therapies in cancer.

## Discussion

Mutations and epitranscriptomic changes in coding regions may alter amino acid sequences and impact protein levels or activity in cancer cells. However, understanding the complex molecular mechanisms of cancers also requires investigating non-coding regions of mRNAs, such as 3’ UTRs, which are crucial for regulating mRNA stability, localization, and translation efficiency (Erson-Bensan 2020). Supporting this, advances in sequencing technologies and computational analysis have revealed that variations in 3’UTR length and RNA modifications within 3’UTRs can significantly impact protein levels, not only in cancer but also in other diseases (Mitschka and Mayr 2022; Liu et al. 2024; Tac et al. 2021; Pinto and Levanon 2019).

Here we used in silico predictions from breast cancer patient RNA-seq data (Han et al. 2015) and determined upregulated editing events commonly detected in breast cancer subtypes. This approach showed an enrichment of upregulated A-to-I editing events in 3’UTRs. Of note, this enrichment is for the commonly edited mRNAs in all breast cancer subtypes, excluding subtype specific events. Additional editing events likely occur in a subtype specific manner and in other regions of mRNAs, as well as in spliced-out introns; however, such events typically go undetected in standard RNA-seq datasets unless the introns are exonized. Furthermore, alternative polyadenylation can generate transcript isoforms with variable 3’UTR lengths. In these cases, editing events that occur in extended 3’UTRs may be misannotated as intergenic regions when compared to canonical gene and isoform structures. As a result, sequencing strategies specifically tailored to capture all A-to-I editing events (Wei et al. 2023), coupled with isoform specific analyses are necessary for a more comprehensive characterization of the editing landscape.

We experimentally validated RNA editing sites in the 3’UTRs of *MDM2*, *GINS1*, and *F11R*. Direct sequencing of RT-PCR products allowed a quantitative comparison of nucleotide counts of cDNA and genomic DNA templated amplicons (Malik et al. 2021). Additional protein-based experiments showed the physical and functional interaction between the selected 3’UTRs and ADAR1.

Of these 3’UTRs, we focused on MDM2 given its role in cancer. MDM2 is best characterized for its E3 ligase activity and its role in the negative regulation of the major tumor suppressor TP53 (reviewed in Yousuf et al., 2025). MDM2 also regulates TP53-independent processes, including cell migration and metastasis (de Queiroz et al. 2024), and targets other proteins such as the microtubule-associated hematopoietic PBX-interaction protein (HPIP), a positive regulator of E2-mediated AKT signaling (Shostak et al. 2014). Although MDM2 gene amplification has been reported in luminal breast cancers (Wege et al. 2022), the role of epitranscriptomic mechanisms in MDM2 regulation remains largely unexplored in breast cancers. Additionally, correlation of high ADAR protein levels with poor relapse free survival in ER (+) breast tumors warrant further investigation (Fig. S6).

In this study, we identified and confirmed 15 distinct A-to-I(G) editing sites within the *MDM2* 3’UTR and showed seven editing sites to have a positive effect on protein levels in a reporter system. Silencing ADAR1 led to a reduction in A-to-I(G) editing across these 15 sites, accompanied by a significant decrease in MDM2 protein levels, underscoring the regulatory impact of RNA editing on MDM2 expression. Moreover, RNA editing ratios were less in normal breast tissue, suggesting a cancer-specific increase in RNA editing. A reporter assay provided initial insights into the functional potential of several *MDM2* RNA editing sites; however, a more comprehensive analysis using full-length constructs, combined with functional assays, would be more informative in determining whether microRNAs and/or other trans-acting factors differentially bind to the edited transcripts. Based on these results, we suggest, 3’UTR editing to be a contributor to increased MDM2 expression in breast cancers and propose that MDM2 protein can be significantly downregulated by ADAR1 inhibition. Of note, ADAR1 inhibition in cancers may have additional advantages because some tumors depend on ADAR1 and A-to-I(G) editing to escape immune surveillance, potentially enabling anticancer therapies with ADAR1 inhibitors (Rehwinkel and Mehdipour 2025).

In addition, most editing sites are within dsRNA regions derived from the transcription of inverted-repeat *Alu* elements (Liu et al. 2024), but *MDM2* 3’UTR editing sites were not surrounded by *Alu* repeats, suggesting the involvement of other sequences and/or other RNA-binding proteins. Proximity biotinylation by CSTF2 TurboID, LC-MS/MS and co-immunoprecipitation results demonstrated the interaction of ADAR1 and CSTF2, a key polyadenylation machinery protein, opening the possibility to better understand ADAR1 presence in 3’UTRs. The interaction between ADAR1 and CSTF2 may have further implications as was suggested in glioblastoma cells (Bahn et al. 2015). In U87MG cells, ADAR1 has been shown to compete with known 3′UTR-binding factors, highlighting how its occupancy—and that of polyadenylation proteins or other RBPs—can be shaped by tissue-specific regulatory contexts.

In closing this study highlights the significance of A-to-I(G) RNA editing in the 3’UTR of *MDM2* and its contribution to increased MDM2 expression in breast cancers. Our findings demonstrate that RNA editing positively influences MDM2 protein levels, with seven specific editing sites enhancing protein expression in a reporter system. The cancer-specific elevation of RNA editing and the reduction of MDM2 levels upon ADAR1 silencing underscore the regulatory role of ADAR1-mediated 3’UTR editing. Additionally, the interaction between ADAR1 and CSTF2 suggests a complex interplay between RNA editing and mRNA processing. Future studies exploring the functional implications of ADAR1-CSTF2 interaction will also be essential for unraveling the complex dynamics and consequences of RNA editing in cancer progression and treatment strategies.

## Electronic supplementary material

Below is the link to the electronic supplementary material.

Supplementary Material 1

Supplementary Material 2

Supplementary Material 3

Supplementary Material 4

## Data Availability

Data is provided within the manuscript or supplementary information files.
