# Supplementary material for: 3’UTR RNA editing driven by ADAR1 modulates MDM2 expression in breast cancer cells
Source: Funct Integr Genomics. 2025 May 17;25(1):103. doi: 10.1007/s10142-025-01611-3 (PMC12085317; doi:10.1007/s10142-025-01611-3)

Figure 3

a

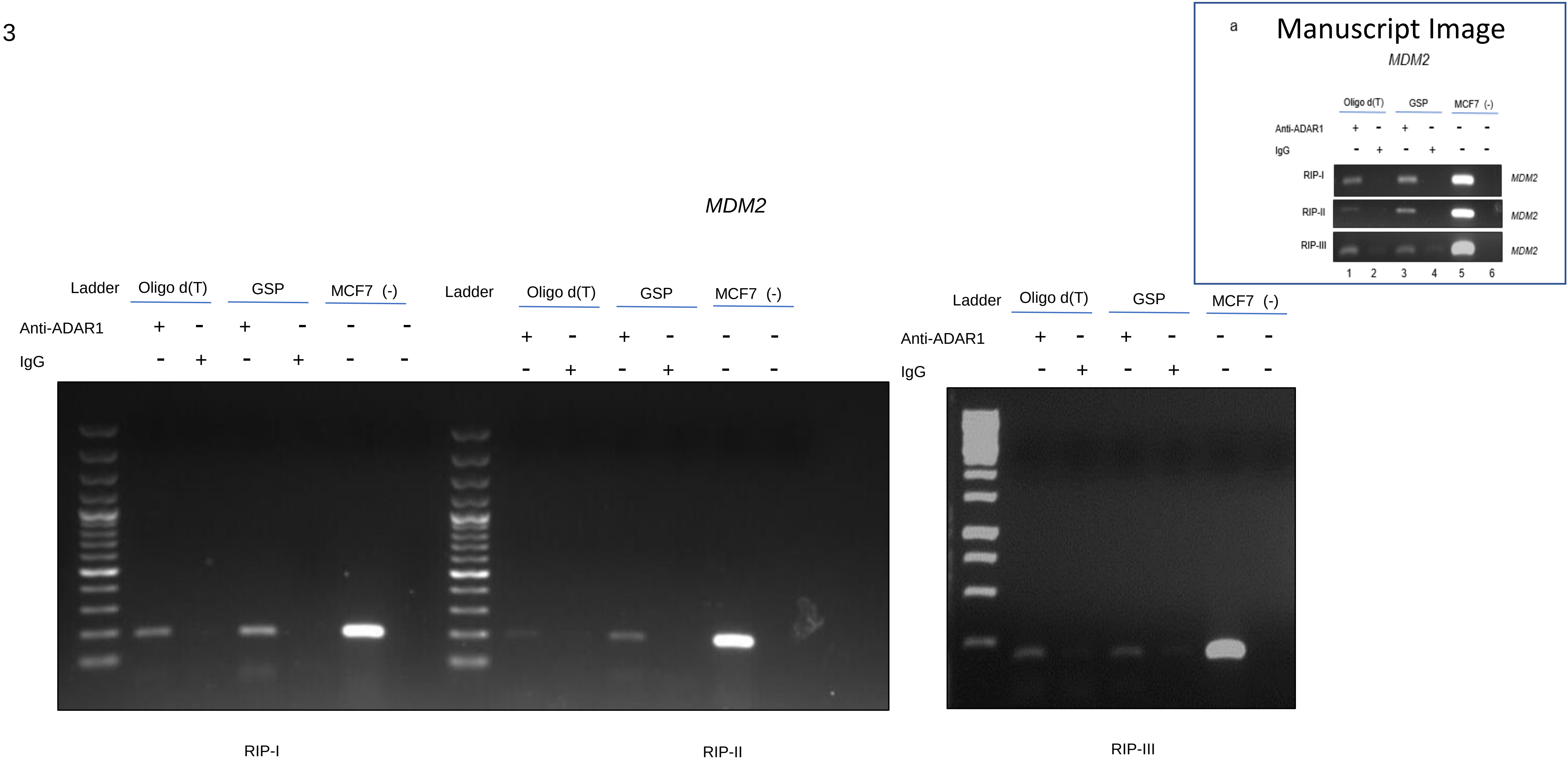

Ladder : GeneRuler 100 bp Plus DNA Ladder

Figure 3

b

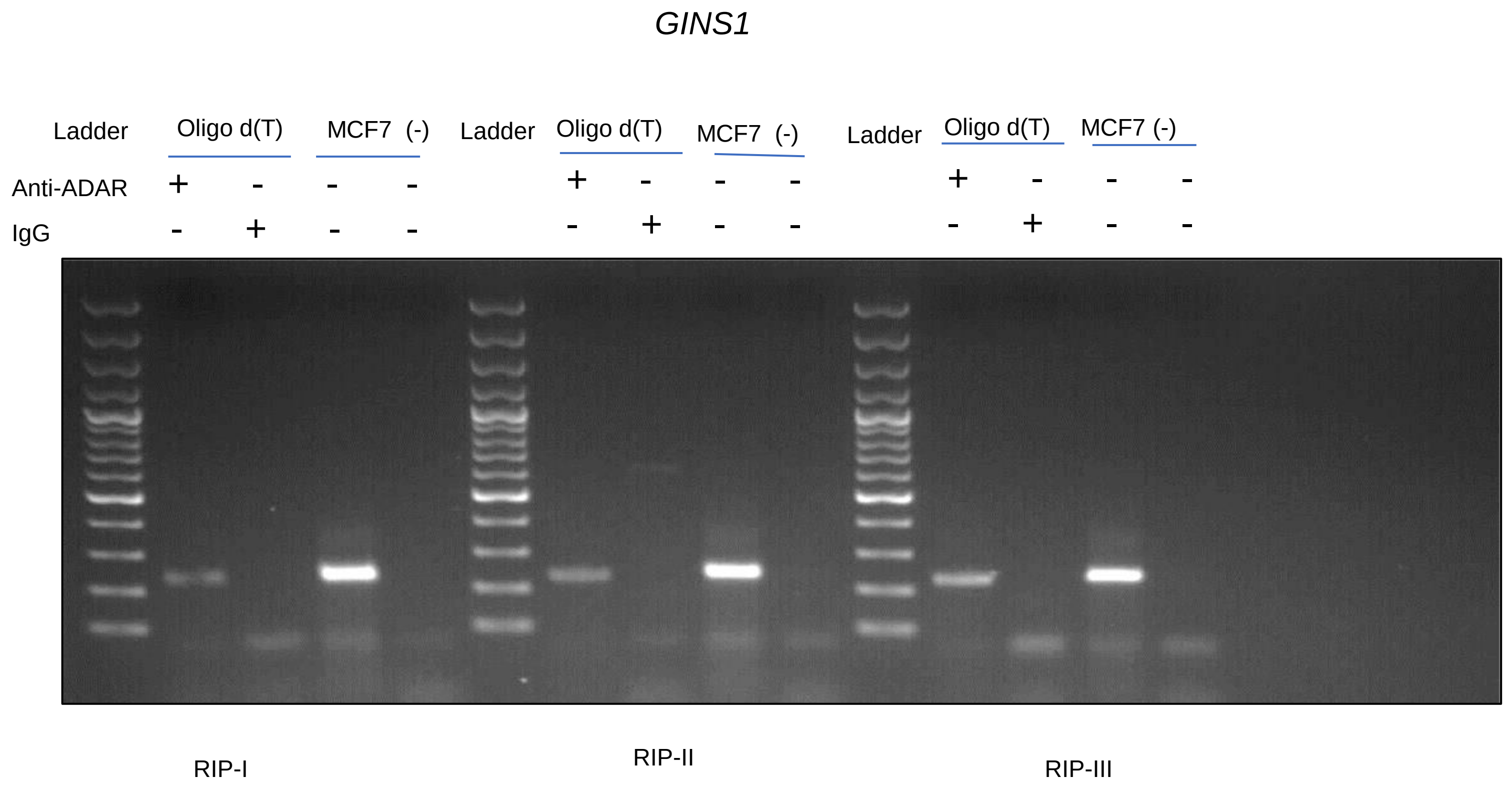

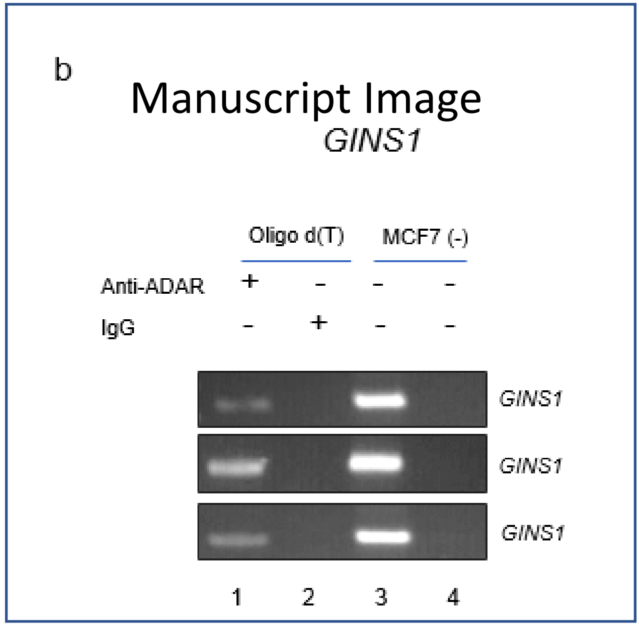

Ladder: GeneRuler 100 bp Plus DNA Ladder

Figure 3

c

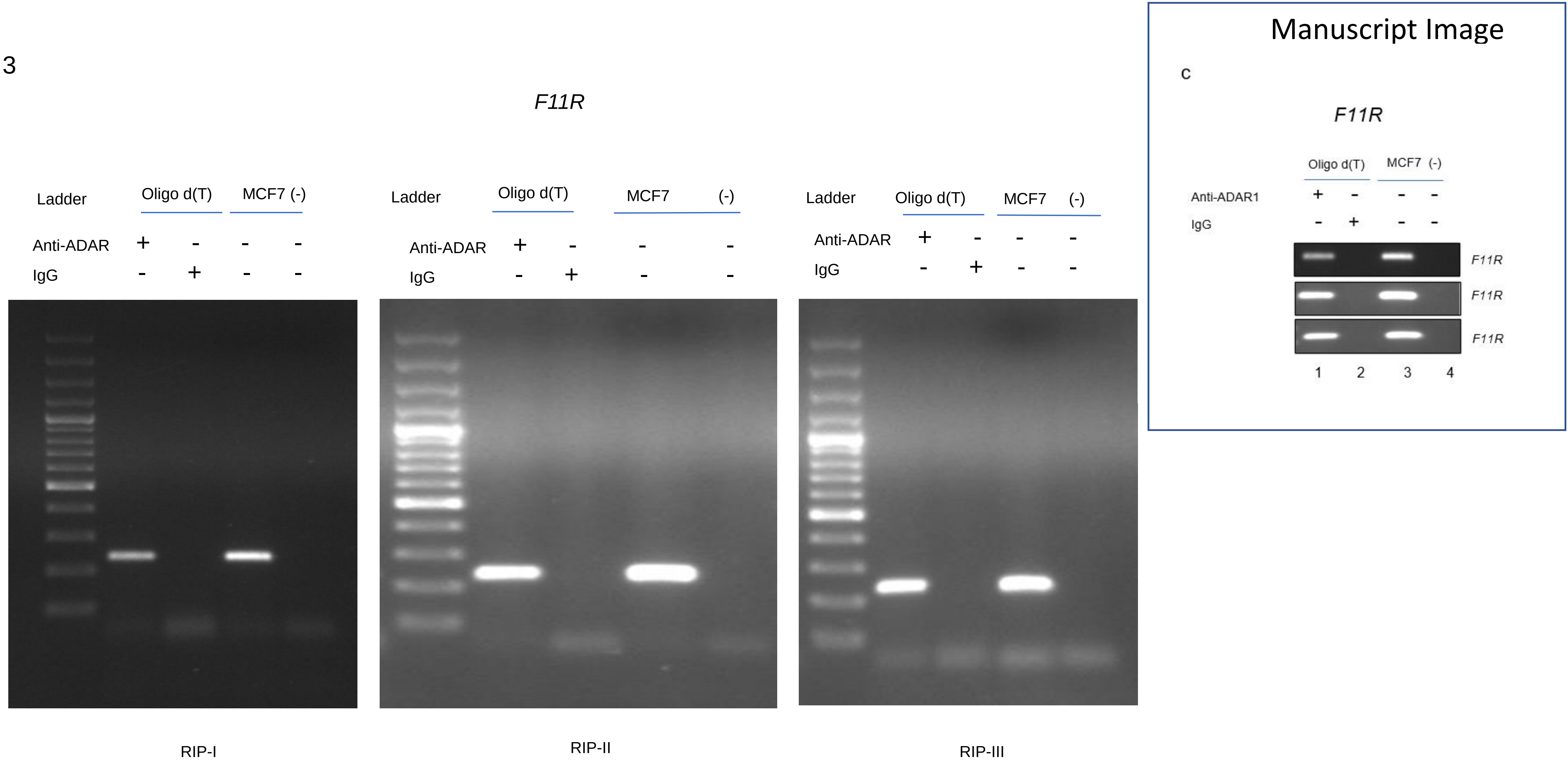

F11R
Ladder
Oligo d(T)
MCF7
(-)
Anti-ADAR
IgG
RIP-I
RIP-II
RIP-III
Manuscript Image
c
F11R
Oligo d(T)
MCF7 (-)
Anti-ADAR1
IgG
F11R
F11R
F11R
1
2
3
4

Figure 4

b

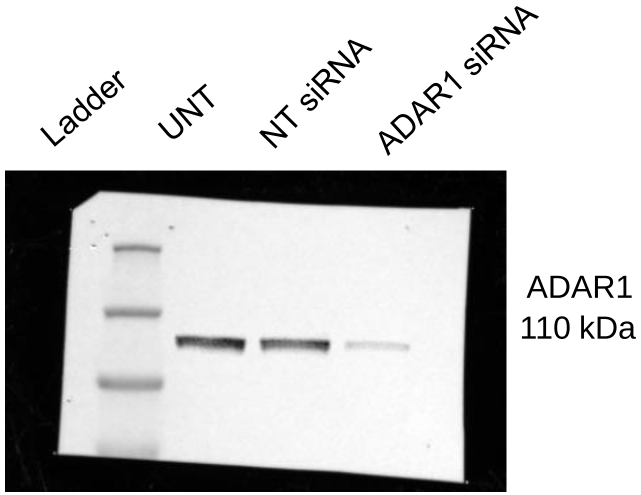

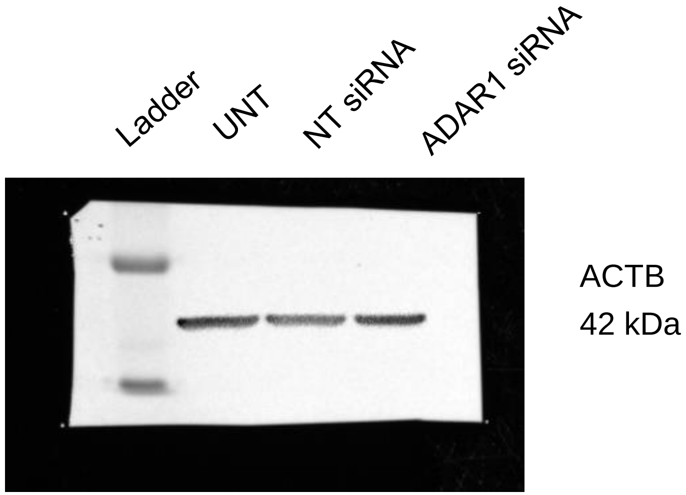

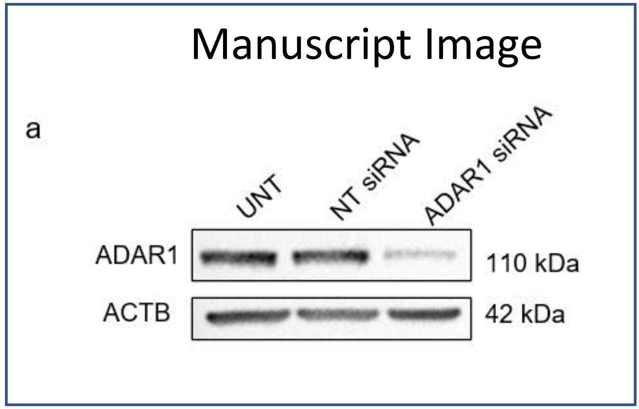

Ladder: Thermo Scientific™ PageRuler™ Plus Prestained Protein Ladder, 10 to 250 kDa

Figure 6

b

Ladder C Ladder UNT NT 24 h 48 h 72 h

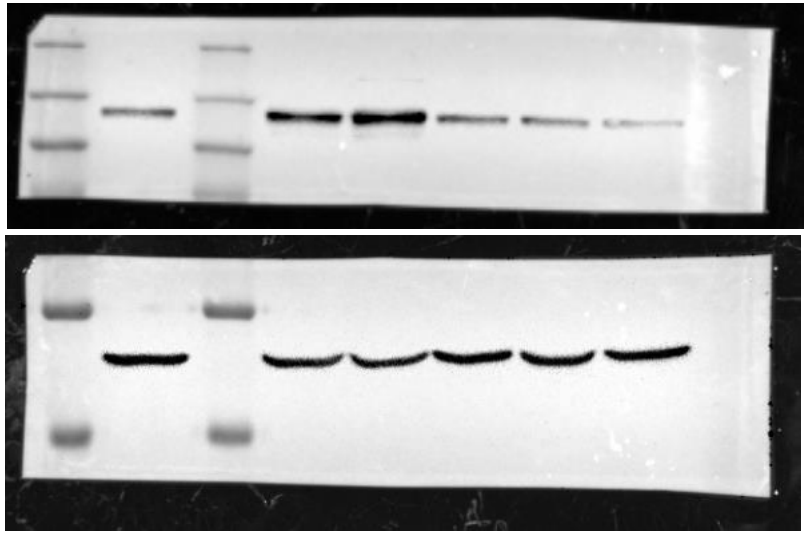

ADAR1
110 kDa

ACTB
42 kDa

Ladder UNT NT 24 h 48 h 72 h

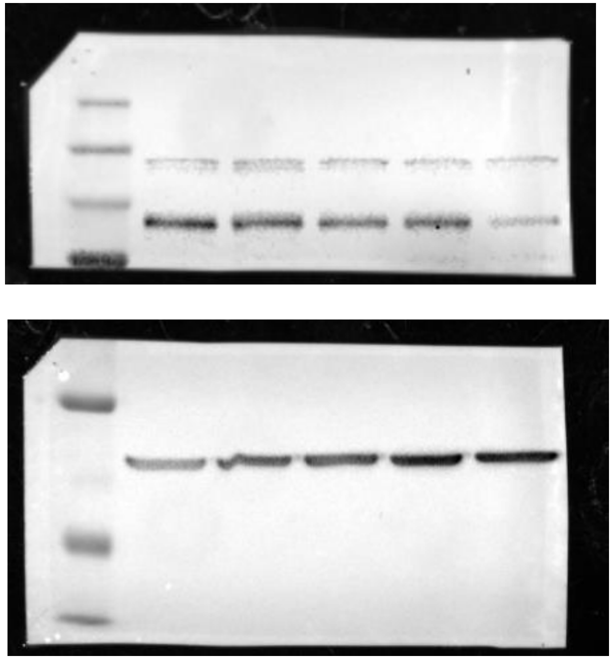

MDM2
90 kDa

ACTB
42 kDa

Manuscript Image

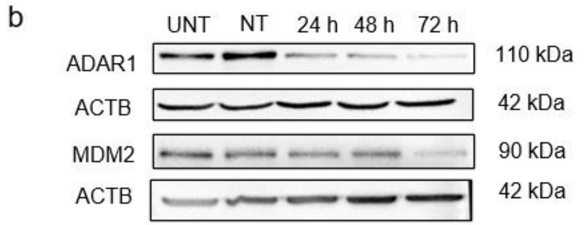

Ladder and wb control lysate lanes were cropped in the manuscript image

Ladder: Thermo Scientific™ PageRuler™ Plus Prestained Protein Ladder, 10 to 250 kDa

Figure 7
a

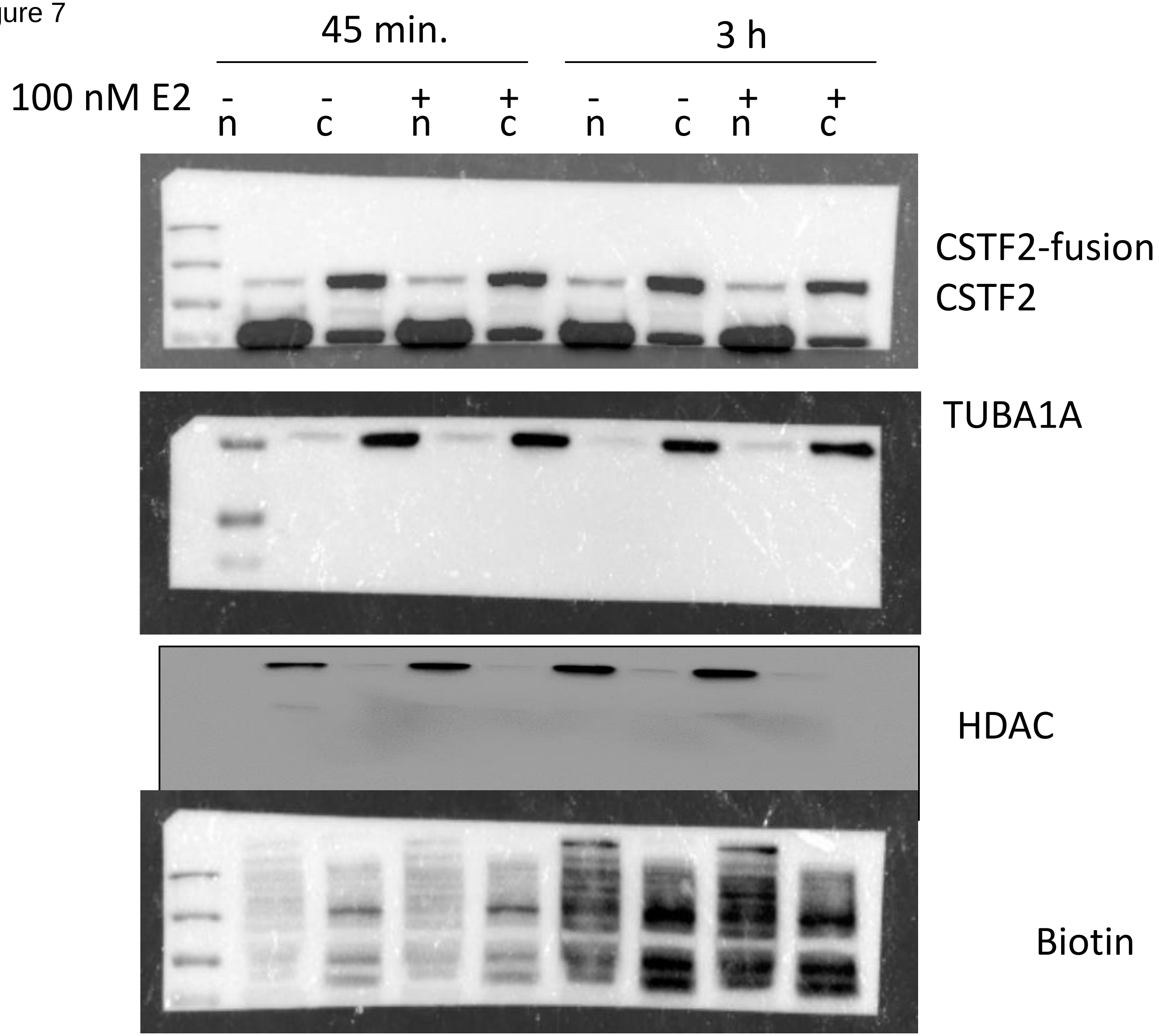

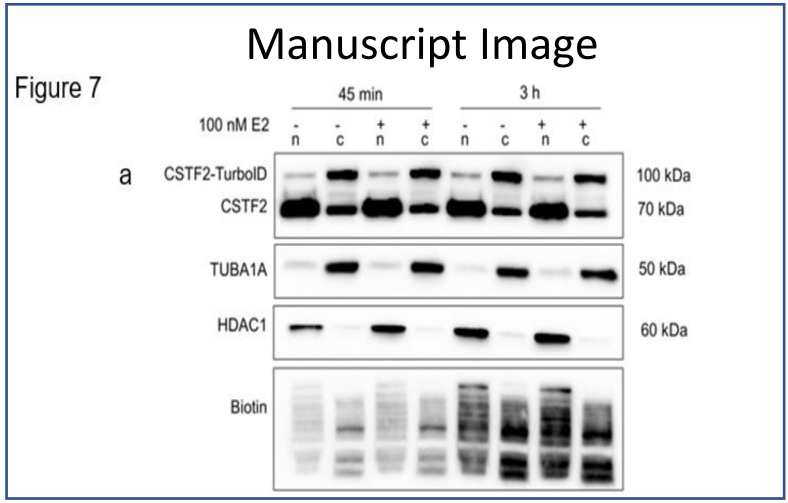

Western Blot image (due to limited biotin pull down lysate concentration, same membrane was cut and probed/reprobed with CSTF2, TUBA1A, HDAC and Biotin antibodies)

Figure 7
d

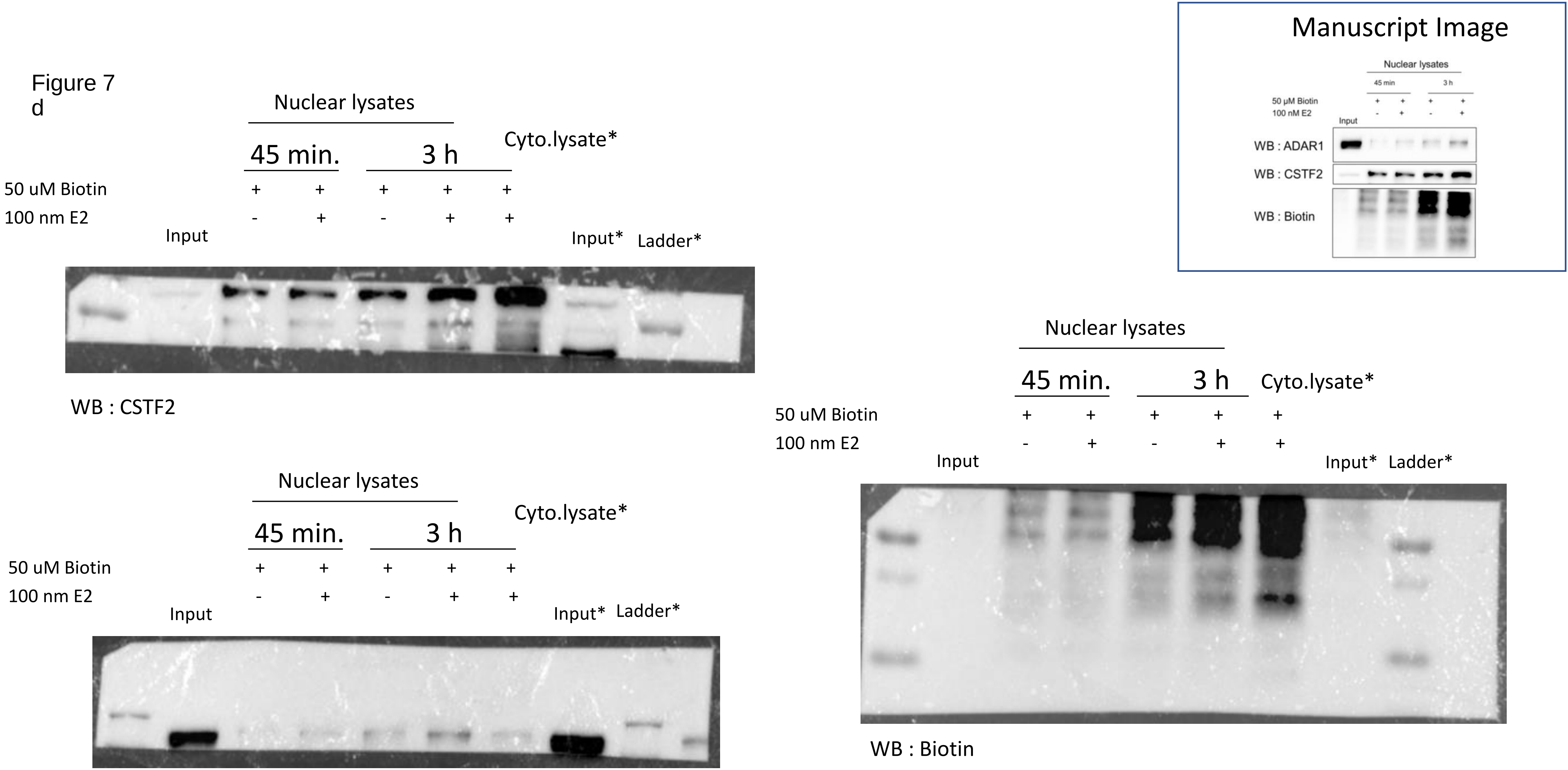

same membrane was cut and probed with CSTF2, ADAR1 and Biotin antibodies

*Cytoplasmic lysate, input and ladder lanes were cropped in the manuscript figure for clarity. Removed input and ladder (last two lanes) are already present in lanes 1-2

Figure 7

e

Manuscript Image

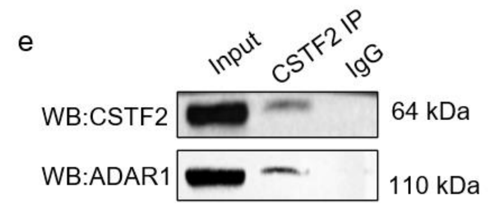

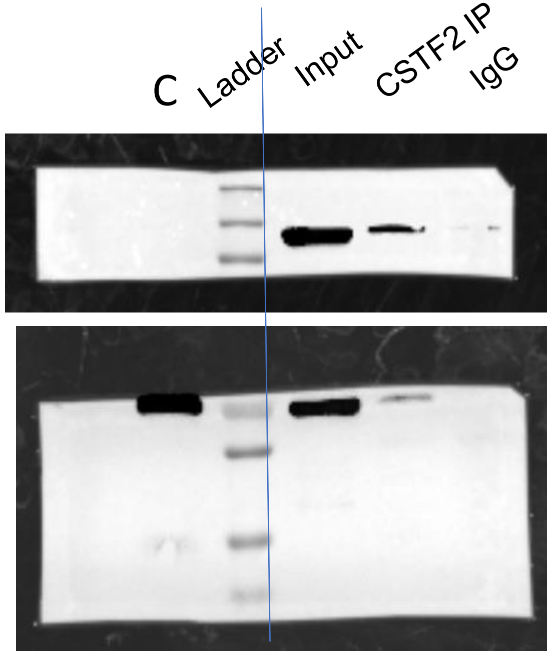

WB:ADAR1
110 kDa

WB:CSTF2
64 kDa

same membrane was cut and probed with CSTF2 and ADAR1 antibodies
Lane 1 was cytoplasmic lysate (c) for wb control (cropped in manuscript image)

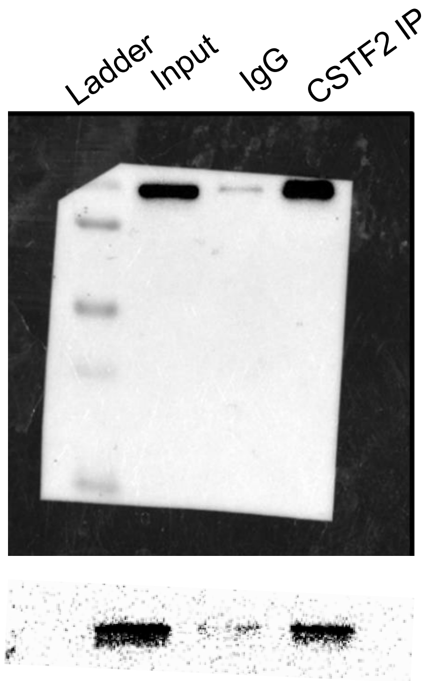

WB:CSTF2

WB:ADAR1

same membrane was cut and probed with CSTF2 and ADAR1 antibodies

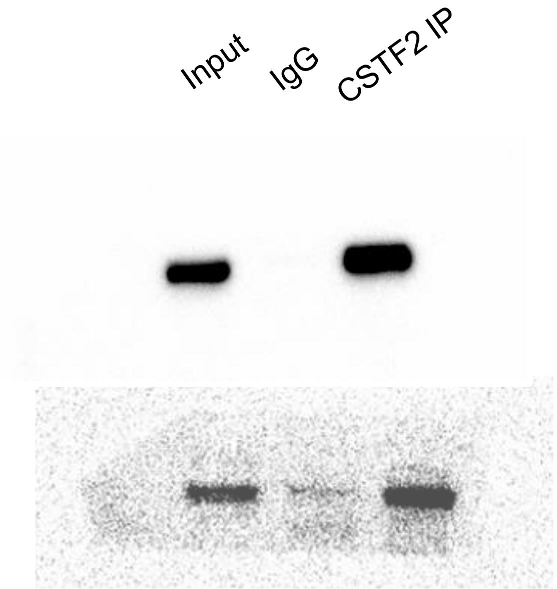

WB:CSTF2

WB:ADAR1

same membrane was cut and probed with CSTF2 and ADAR1 antibodies

(In supp. Figures)

Ladder: Thermo Scientific™ PageRuler™ Plus Prestained Protein Ladder, 10 to 250 kDa

Figure 8

Manuscript Image

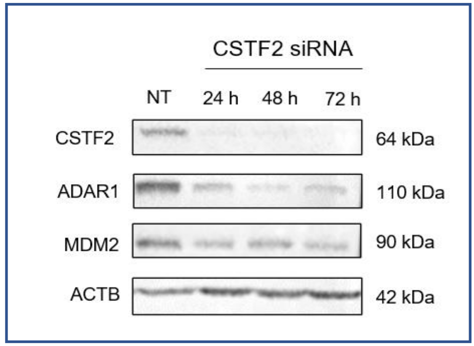

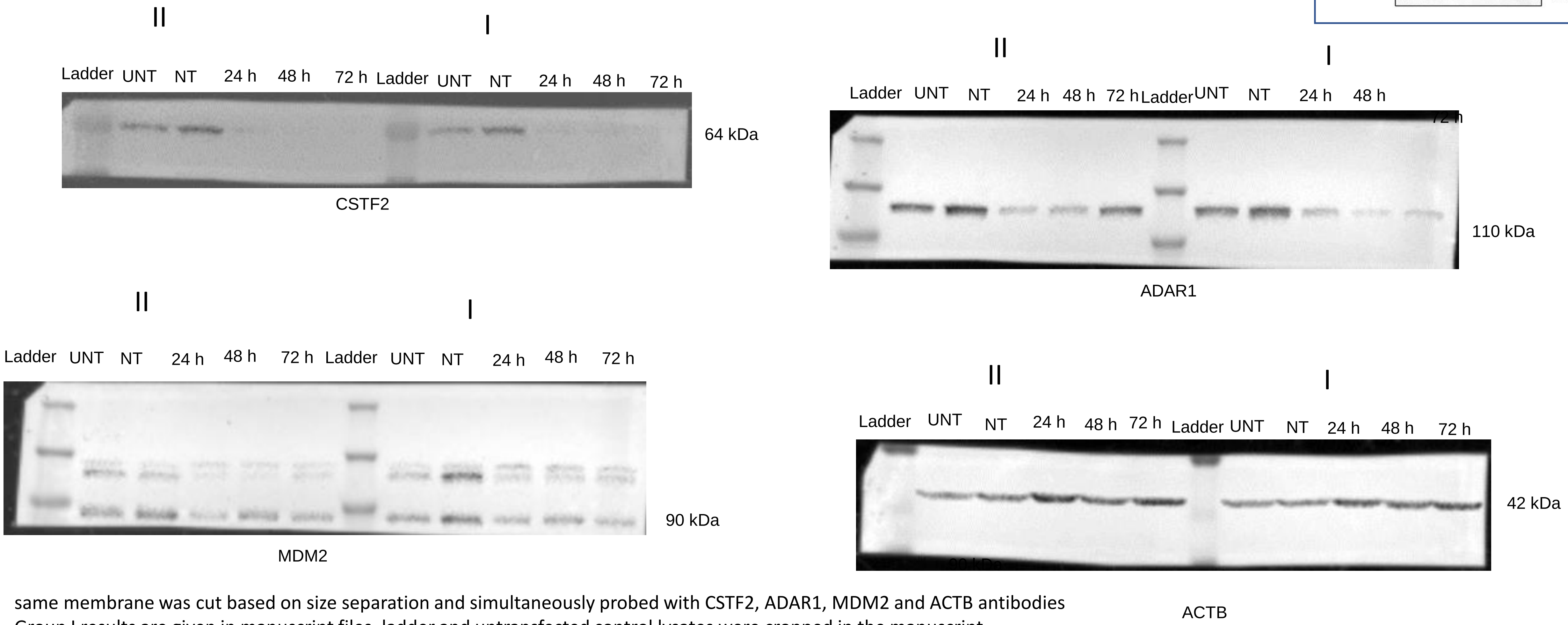

same membrane was cut based on size separation and simultaneously probed with CSTF2, ADAR1, MDM2 and ACTB antibodies
Group I results are given in manuscript files, ladder and untransfected control lysates were cropped in the manuscript.

Ladder: Thermo Scientific™ PageRuler™ Plus Prestained Protein Ladder, 10 to 250 kDa

Figure S3

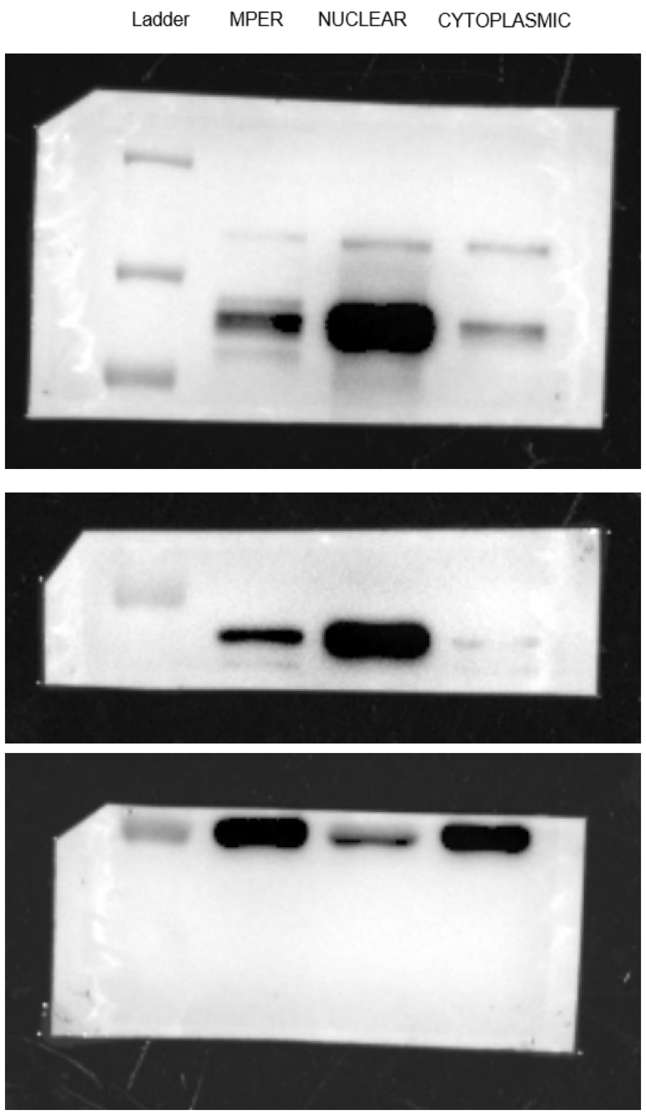

Manuscript Image (Supp)

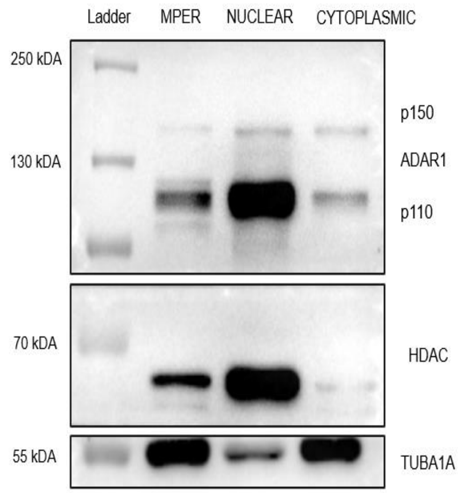

Supplement: Supplementary file 1 — Supplementary Material 1 [file 10142_2025_1611_MOESM1_ESM.pdf]
