# Supplementary material for: 3’UTR RNA editing driven by ADAR1 modulates MDM2 expression in breast cancer cells
Source: Funct Integr Genomics. 2025 May 17;25(1):103. doi: 10.1007/s10142-025-01611-3 (PMC12085317; doi:10.1007/s10142-025-01611-3)
Supplement: Supplementary file 2 — Supplementary Material 2 [file 10142_2025_1611_MOESM2_ESM.pdf]

Supplementary Figures

a

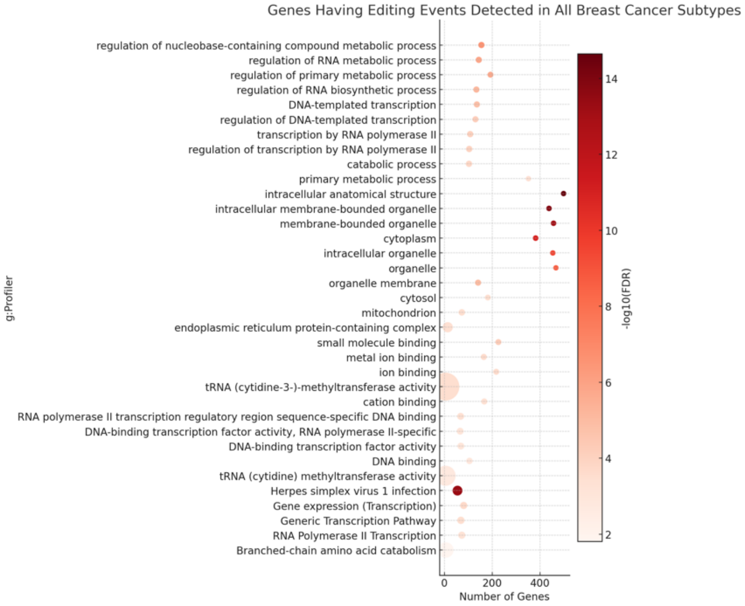

b

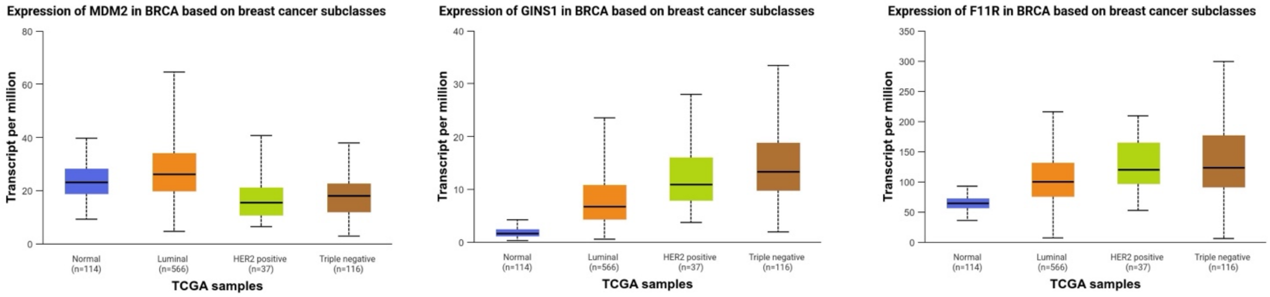

Comparison	Statistical significance
**Normal-vs-Luminal**	**2.98880000004775E-06**
Normal-vs-HER2 Positive	5.377600E-01
Normal-vs-TNBC	1.602950E-01
**Luminal-vs-HER2 Positive**	**1.990120E-02**
**Luminal-vs-TNBC**	**2.99670000036834E-07**
HER2 Positive-vs-TNBC	8.010000E-01

Comparison	Statistical significance
**Normal-vs-Luminal**	**<1E-12**
**Normal-vs-HER2 Positive**	**2.30926389122033E-12**
**Normal-vs-TNBC**	**<1E-12**
**Luminal-vs-HER2 Positive**	**1.075880E-02**
**Luminal-vs-TNBC**	**8.86340001393648E-10**
**HER2 Positive-vs-TNBC**	**1.296360E-02**

Comparison	Statistical significance
**Normal-vs-Luminal**	**1.62436730732907E-12**
**Normal-vs-HER2 Positive**	**3.97629706938574E-11**
**Normal-vs-TNBC**	**1.62447832963153E-12**
Luminal-vs-HER2 Positive	6.561800E-02
**Luminal-vs-TNBC**	**3.73490000000753E-05**
HER2 Positive-vs-TNBC	2.299800E-01

Figure S1. a. Functional enrichment profile for RNA editing candidates. The x-axis indicates the number of input genes associated with each term. Dot size reflects the gene ratio (input term coverage), and color represents $-\log_{10}$ FDR corrected for multiple testing using g:SCS by g:Profiler, b. Expression of *MDM2*, *GINS1* and *F11R* in TCGA breast cancer dataset. Data retrieved from UALCAN (https://ualcan.path.uab.edu/).

a. *MDM2* 3’UTR (MCF7)

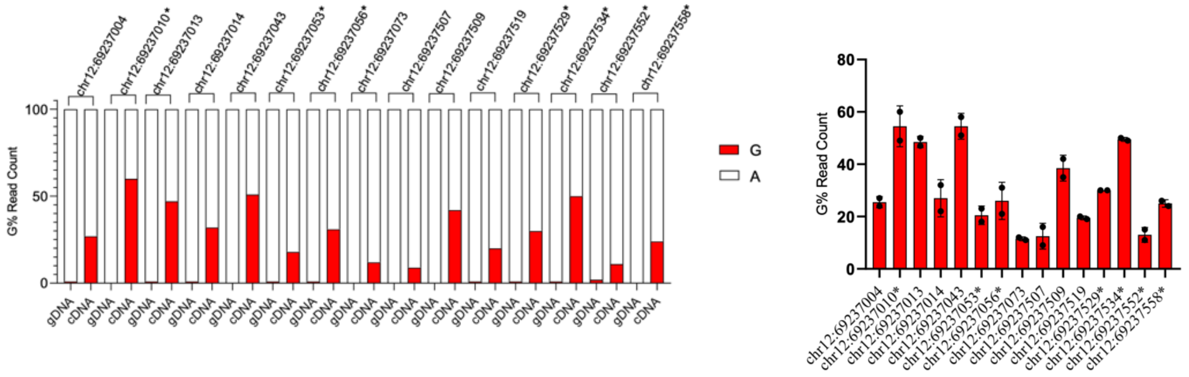

b. *MDM2* 3’UTR (MDA-MB-231)

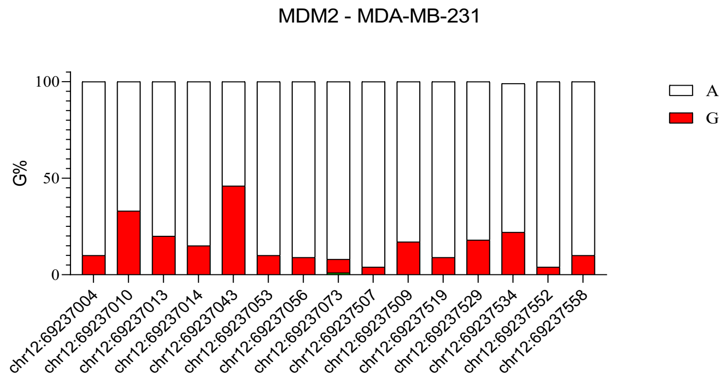

c. *GINS1* 3’UTR (MDA-MB-231)

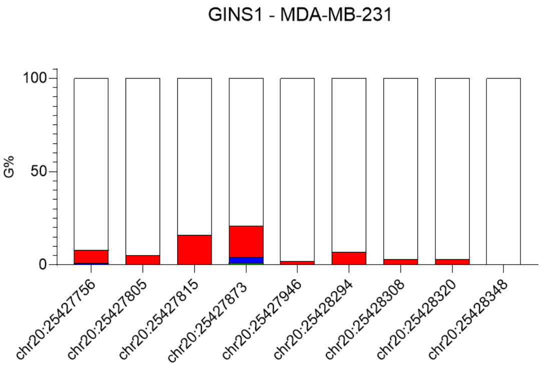

d. *F11R* 3’UTR (MDA-MB-231)

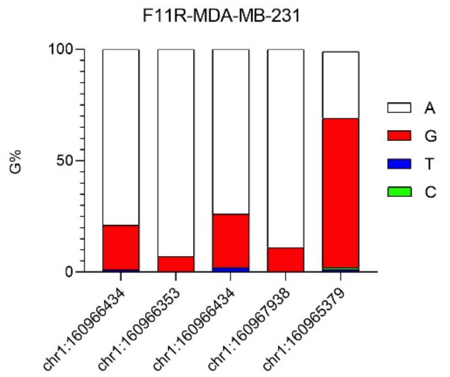

e. *MDM2* 3’UTR NT siRNA and ADAR1 siRNA transfected MCF7 cells

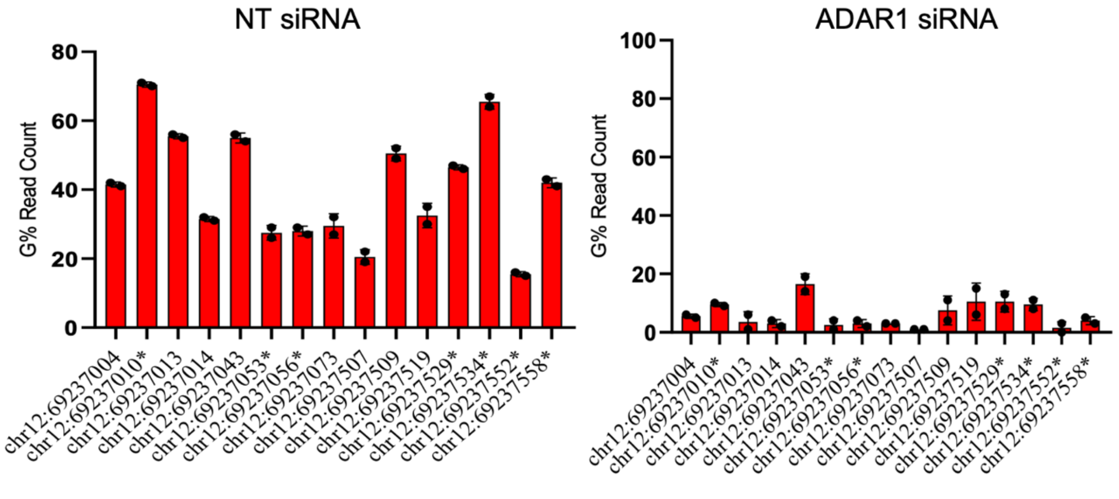

Figure S2. a. 3’UTR A-to-I(G) editing of *MDM2* 3’UTR in MCF7 cells. gDNA and cDNA templated PCR amplicons were sequenced and G% values were calculated based on NGS read counts (second biological replicate). The A-to-I(G) editing percentages for the two independent NGS analysis are shown on the right, b. *MDM2,* c. *GINS1,* d. *F11R* 3’UTR A-to-I(G) editing in MDA-MB-231 cells, e) 3’UTR A-to-I(G) editing in NT (non-targeting) and ADAR1 siRNA transfected MCF7 cells (n=2).

a

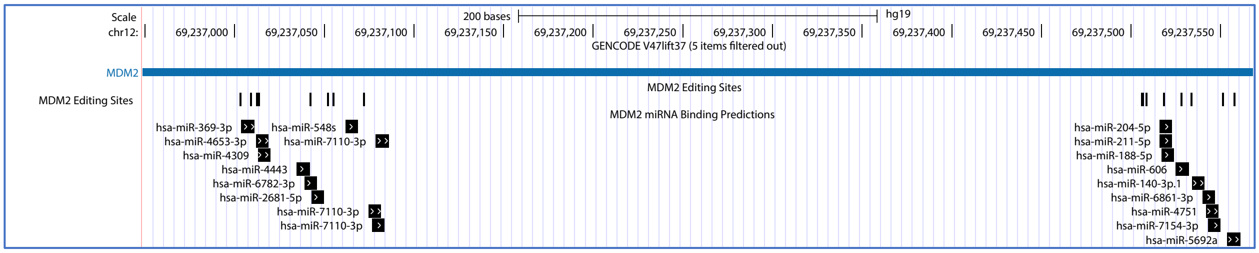

b

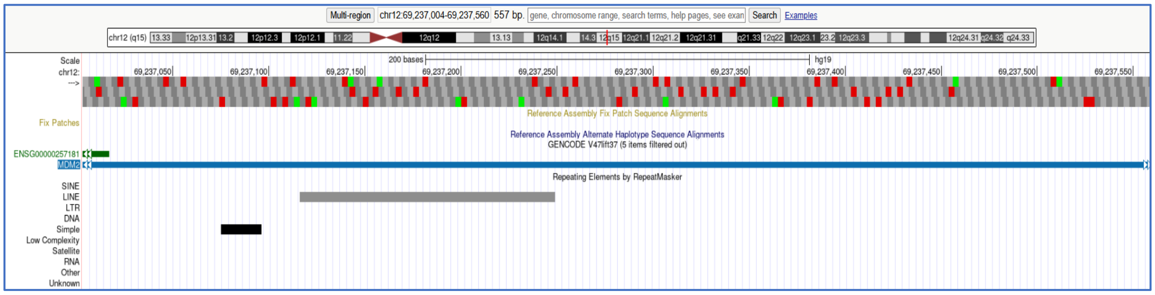

Figure S3. a. Predicted miRNA binding sites and confirmed editing sites on MDM2 3'UTR are shown. Custom BED files were generated based on predicted miRNA target sites for MDM2 by TargetScanHuman version 7.2, and were uploaded to the UCSC Genome Browser (hg19 assembly). Poorly conserved miRNA predictions with a context++ score percentile ≥70 were selected to ensure high confidence, b. *MDM2* RNA editing regions (chr12:69,237,004-69,237,560) were visualized in UCSC Genome Browser (GRCh37/hg19). RepeatMasker was used to investigate the presence of repeat elements. There were no Alu elements around the A-to-I(G) editing sites on the 3'UTR of *MDM2*. (GA) repeats (black) and L1MA5A elements (Family: L1, Class: LINE) (gray) are shown.

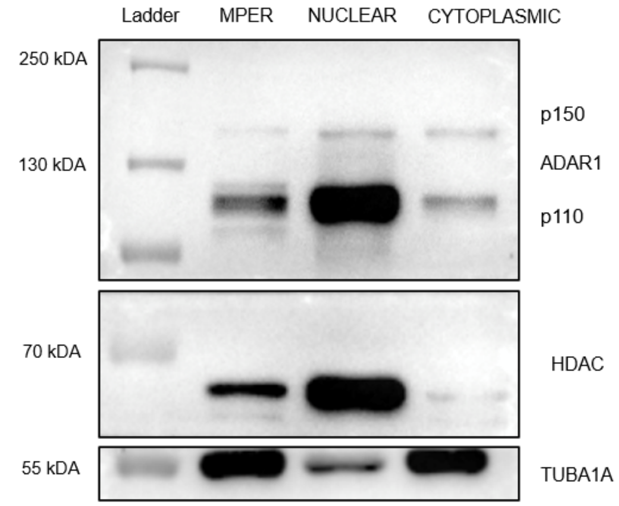

Figure S4. ADAR1 expression in nuclear and cytoplasmic fractions of MCF7 protein lysates.

MPER (Mammalian Protein Extraction Reagent, Thermo Fisher Scientific, 78835) was used to extract total protein. Nuclear and cytoplasmic lysates (25 µg) were isolated using NEPER Nuclear and Cytoplasmic Reagents (Thermo Fisher Scientific, 78835). Same blots were hybridized with HDAC (SantaCruz, sc-81598) antibody for nuclear protein and with TUBA1A (Cell Signaling, 2144) antibody for cytoplasmic lysate verification.

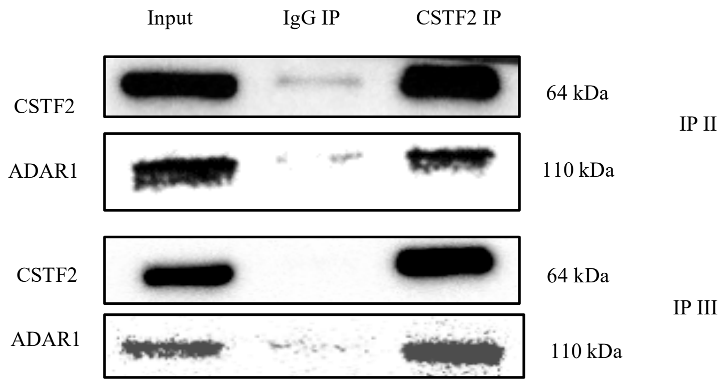

Figure S5. Nuclear extracts (500 μg) of MCF7 cells were subjected to Co-IP with CSTF2 (Santa Cruz, sc-398862) or isotype-matched IgG (Santa Cruz, sc-2025). Input was 25 μg nuclear lysate. Immunoprecipitated proteins were then subjected to immunoblotting using CSTF2 (Santa Cruz sc-398862) or ADAR1 (Cell Signaling, 14175) antibodies.

a

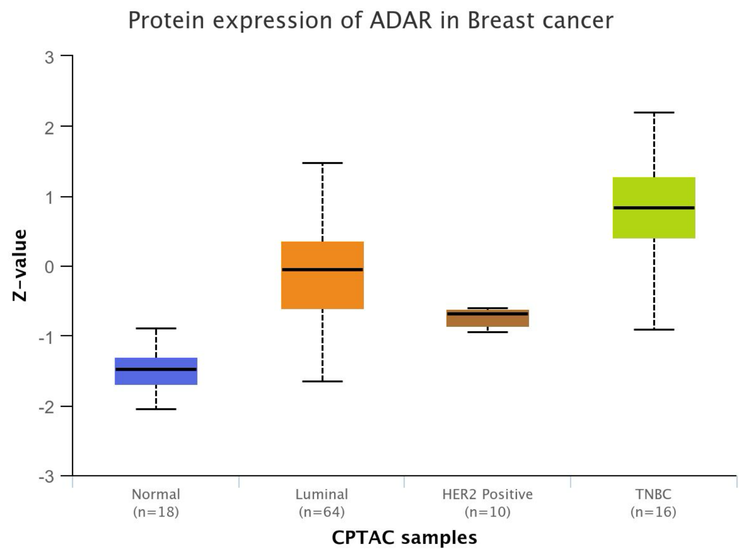

Comparison	Statistical significance
Normal-vs-Luminal	2.12539154176067E-14
Normal-vs-HER2 Positive	1.466368E-02
Normal-vs-TNBC	1.88113967387158E-07
Luminal-vs-HER2 Positive	1.085303E-01
Luminal-vs-TNBC	1.824585E-02
HER2 Positive-vs-TNBC	4.140728E-03

b c

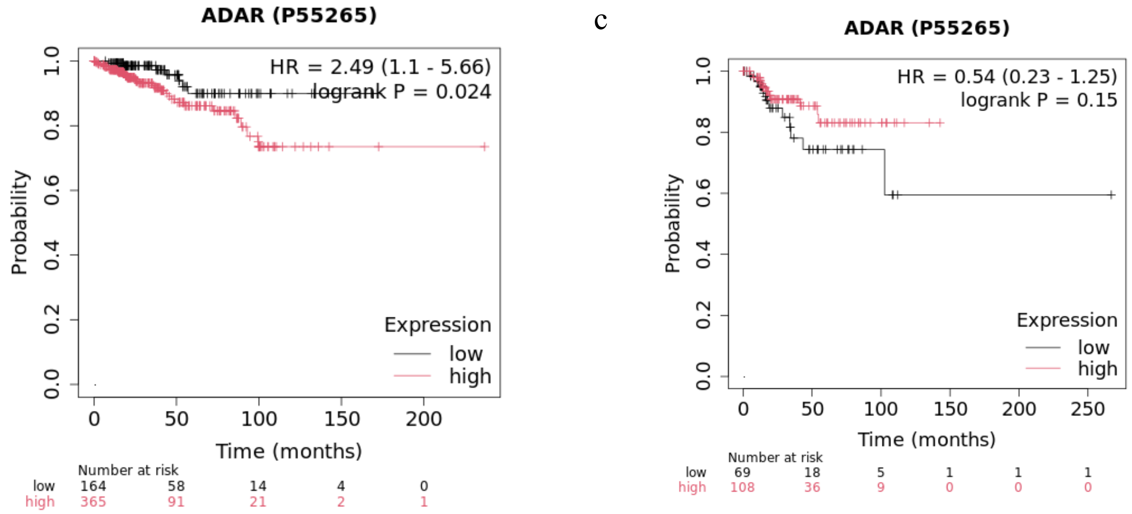

Figure S6. a. ADAR protein expression in major breast cancer subtypes (luminal, Her2+, TNBC) compared to normal tissue. Analysis of CPTAC data was retrieved from UALCAN database (https://ualcan.path.uab.edu/), b. Kaplan-Meier analysis of ADAR protein levels and relapse free survival in ER (+) and (c) ER (-) breast tumors of the TCGA-RPPA dataset (Data was retrieved from https://kmplot.com/) (ADAR Uniprot ID: P55265, Auto select best cutoff: percentile).

a

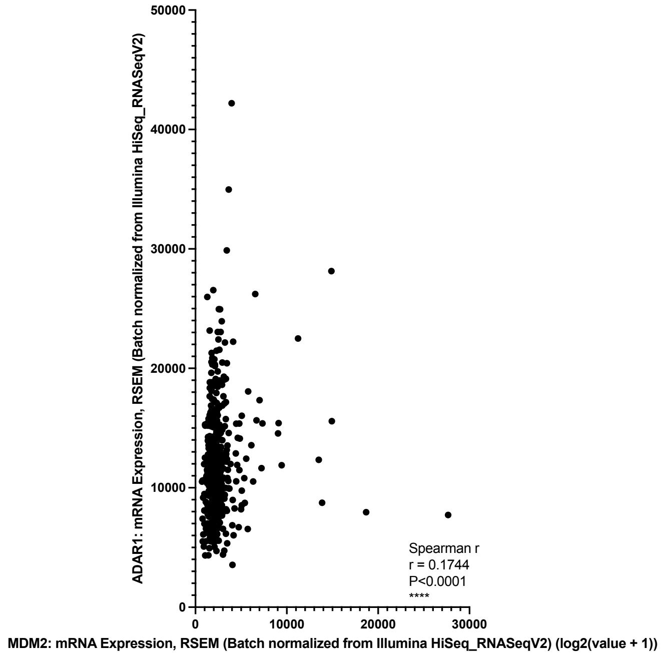

b

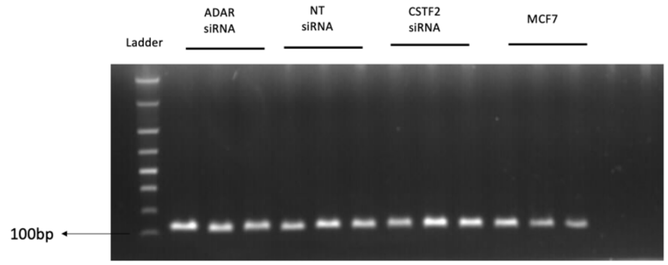

Fig. S7. a. Lack of correlation between *MDM2* and *ADAR1* mRNA expression in Luminal A breast cancer samples (TCGA, PanCancer Atlas). Expression values were obtained as RSEM (RNA-Seq by Expectation Maximization), batch-normalized from Illumina HiSeq_RNASeqV2. Spearman's rank correlation test revealed lack of correlation (r = 0.1744, P < 0.0001). Correlation analysis was performed using GraphPad Prism, b. *MDM2* RT-PCR in MCF7 cells transfected with NT siRNA, ADAR1 siRNA and CSTF2 siRNA for 72 hours. RNA was isolated and cDNA was synthesized as described in the methods section.
