# Supplementary material for: 3’UTR RNA editing driven by ADAR1 modulates MDM2 expression in breast cancer cells
Source: Funct Integr Genomics. 2025 May 17;25(1):103. doi: 10.1007/s10142-025-01611-3 (PMC12085317; doi:10.1007/s10142-025-01611-3)

**Supplementary Tables**

**Table S2.** RT-PCR primers

| Primers | Sequence (5’🡪3’) |
| --- | --- |
| MDM2_F | AGAGCCGAATAAGGTTTGCC |
| MDM2_R | GCTGTCACTGCCTCCATATATCA |
| GINS1_F | GCTTGTGGCTATGGGGTGAT |
| GINS1_R | ATGTGGTACACTGGGCCAGCAC |
| F11R_F | CAGTCAAGGGTCACATGCCA |
| F11R_R | TCCTCAGGCCCCAAGAAACAT |

**Table S3.** Positions of editing sites for MDM2, GINS1 and F11R

| **Gene** | **Position** | **Gene** | **Position** | **Gene** | **Position** |
| --- | --- | --- | --- | --- | --- |
| MDM2 | chr12:69237004 | GINS1 | chr20:25427756 | F11R | chr1:160967938 |
| MDM2 | chr12:69237010 | GINS1 | chr20:25427805 | F11R | chr1:160965379 |
| MDM2 | chr12:69237013 | GINS1 | chr20:25427815 | F11R | chr1:160966352 |
| MDM2 | chr12:69237014 | GINS1 | chr20:25427873 | F11R | chr1:160966434 |
| MDM2 | chr12:69237043 | GINS1 | chr20:25427946 | F11R | chr1:160966353 |
| MDM2 | chr12:69237053 | GINS1 | chr20:25428294 | F11R | chr1:160965434 |
| MDM2 | chr12:69237056 | GINS1 | chr20:25428308 | F11R | chr1:160966354 |
| MDM2 | chr12:69237073 | GINS1 | chr20:25428320 |  |  |
| MDM2 | chr12:69237507 | GINS1 | chr20:25428348 |  |  |
| MDM2 | chr12:69237509 |  |  |  |  |
| MDM2 | chr12:69237519 |  |  |  |  |
| MDM2 | chr12:69237529 |  |  |  |  |
| MDM2 | chr12:69237534 |  |  |  |  |
| MDM2 | chr12:69237552 |  |  |  |  |
| MDM2 | chr12:69237558 |  |  |  |  |

**Table S4.** *MDM2* editing sites confirmed in MCF7 cells were listed in DARNED (https://darned.ucc.ie/).

| Location | Breast  Cancer | Lymphoblastoid  Cell | Cerebellum | U87MG | MCF7 |
| --- | --- | --- | --- | --- | --- |
| chr12:69237004 | + | + | + | + | + |
| chr12:69237010 | + | + |  | + | + |
| chr12:69237013 | + | + |  |  | + |
| chr12:69237014 |  | + |  |  | + |
| chr12:69237043 |  | + |  | + | + |
| chr12:69237053 | + | + |  |  | + |
| chr12:69237056 |  | + |  |  | + |
| chr12:69237073 |  | + |  |  | + |
| chr12:69237507 |  | + |  |  | + |
| chr12:69237509 |  | + |  |  | + |
| chr12:69237519 |  |  |  | + | + |
| chr12:69237529 |  |  |  |  | + |
| chr12:69237534 |  | + |  | + | + |
| chr12:69237552 |  | + |  |  | + |
| chr12:69237558 |  |  |  |  | + |

**Table S5.** *GINS1* editing sites confirmed in MCF7 cells were listed in DARNED (https://darned.ucc.ie/).

| Location | Breast Cancer | Lymphoblastoid Cell | Venous  Blood | U87MG | MCF7 |
| --- | --- | --- | --- | --- | --- |
| chr20:25427756 | + |  |  |  | + |
| chr20:25427805 |  | + |  |  | + |
| chr20:25427815 |  | + |  |  | + |
| chr20:25427873 |  | + |  |  | + |
| chr20:25427946 |  | + |  |  | + |
| chr20:25428294 |  | + | + | + | + |
| chr20:25428308 |  | + |  |  | + |
| chr20:25428320 |  | + |  |  | + |
| chr20:25428348 |  | + |  |  | + |

**Table S6.** *F11R* editing sites confirmed in MCF7 cells were listed in DARNED (https://darned.ucc.ie/).

| Location | Breast Cancer | Lymphoblastoid Cell | MCF7 |
| --- | --- | --- | --- |
| chr1:160967938 |  | + | + |
| chr1:160966352 |  | + | + |
| chr1:160966353 |  | + | + |
| chr1:160966434 | + | + | + |

**Table S7**. Comparison of G% reads in MCF7 and normal breast tissue

| **Location** | **G % MCF7** | **G% normal breast** |
| --- | --- | --- |
| chr12:69237004 | 24 | 26 |
| chr12:69237010 | 49 | 31 |
| chr12:69237013 | 50 | 26 |
| chr12:69237014 | 22 | 13 |
| chr12:69237043 | 58 | 34 |
| chr12:69237053 | 23 | 13 |
| chr12:69237056 | 21 | 12 |
| chr12:69237073 | 11 | 11 |
| chr12:69237507 | 16 | 12 |
| chr12:69237509 | 35 | 12 |
| chr12:69237519 | 19 | 9 |
| chr12:69237529 | 30 | 28 |
| chr12:69237534 | 49 | 37 |
| chr12:69237552 | 15 | 10 |
| chr12:69237558 | 26 | 9 |

**Table S8.** SaintScores of biotinylated nuclear proteins (E2/Ethanol treated) MCF7 cells transfected with CSTF2-TurboID fusion.


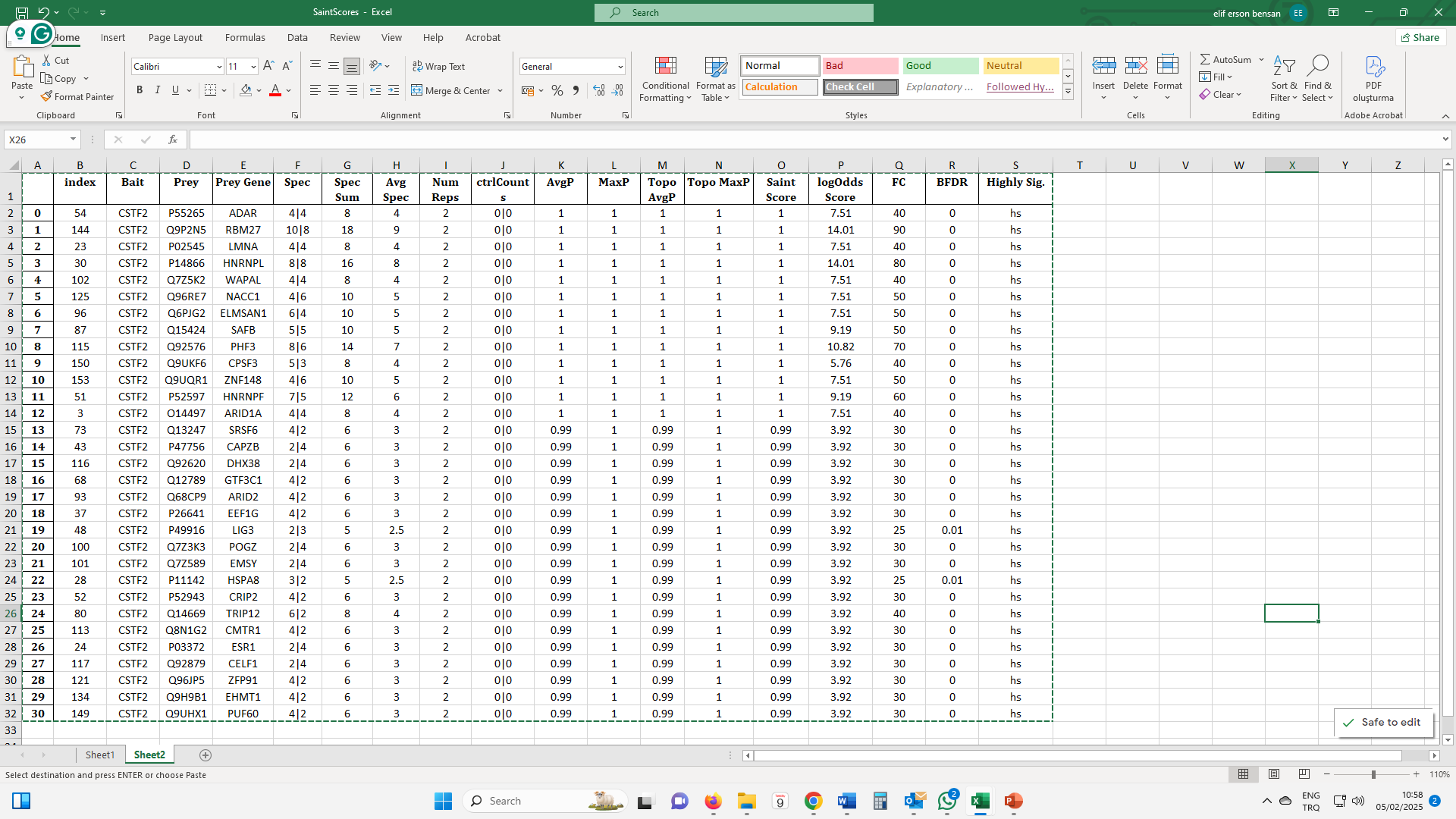

Supplement: Supplementary file 3 — Supplementary Material 3 [file 10142_2025_1611_MOESM3_ESM.docx]
